# A highly selective biosynthetic pathway to non-natural C_50_ carotenoids assembled from moderately selective enzymes

**DOI:** 10.1038/ncomms8534

**Published:** 2015-07-14

**Authors:** Maiko Furubayashi, Mayu Ikezumi, Shinichi Takaichi, Takashi Maoka, Hisashi Hemmi, Takuya Ogawa, Kyoichi Saito, Alexander V Tobias, Daisuke Umeno

**Affiliations:** 1 Department of Applied Chemistry and Biotechnology, Chiba University, Chiba 263-8522, Japan; 2 Department of Biology, Nippon Medical School, Musashino, Tokyo 180-0023, Japan; 3 Research Institute for Production Development, Kyoto 606-0805, Japan; 4 Department of Applied Molecular Biosciences, Nagoya University, Nagoya 464-8601, Japan; 5 DuPont Industrial Biosciences, Experimental Station, Wilmington, Delaware 19803, USA; 6 Precursory Research for Embryonic Science and Technology (PRESTO), Japan Science and Technology Agency (JST), Saitama 332-0012, Japan

## Abstract

Synthetic biology aspires to construct natural and non-natural pathways to useful compounds. However, pathways that rely on multiple promiscuous enzymes may branch, which might preclude selective production of the target compound. Here, we describe the assembly of a six-enzyme pathway in *Escherichia coli* for the synthesis of C_50_-astaxanthin, a non-natural purple carotenoid. We show that by judicious matching of engineered size-selectivity variants of the first two enzymes in the pathway, farnesyl diphosphate synthase (FDS) and carotenoid synthase (CrtM), branching and the production of non-target compounds can be suppressed, enriching the proportion of C_50_ backbones produced. We then further extend the C_50_ pathway using evolved or wild-type downstream enzymes. Despite not containing any substrate- or product-specific enzymes, the resulting pathway detectably produces only C_50_ carotenoids, including ∼90% C_50_-astaxanthin. Using this approach, highly selective pathways can be engineered without developing absolutely specific enzymes.

## Abstract (web summary)

Synthetic engineering of complex pathways is often hindered by pathway branching and generation of non-target compounds. Here, the authors show that by judicious combination of moderately selective enzyme variants, a non-natural C50 carotenoid can be generated in bacteria with minimal production of unwanted compounds.

## Graphical abstract

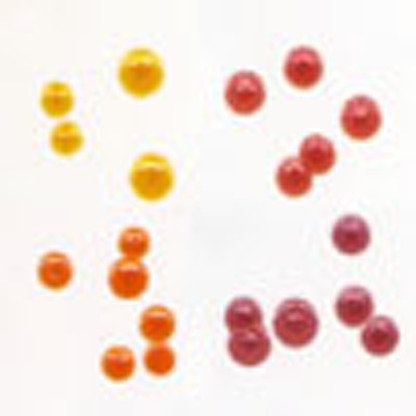

One of the goals of synthetic biology is the engineering of organisms to synthesize useful chemicals, some of which may not be produced in nature^1^. Recent studies have begun to explore the space of novel metabolites by mixing, matching and laboratory-evolving biosynthetic genes from diverse sources in bacteria and yeasts^2^,^3^,^4^,^5^,^6^. In extending natural pathways in novel directions, pathway engineers usually rely on the promiscuous activities (the ability to accept alternative substrates and/or synthesize alternative products) of recruited or mutated enzymes^4^,^5^,^6^,^7^,^8^,^9^,^10^. Unfortunately, the use of such promiscuous enzymes, especially in succession, often generates pathway branch points to numerous byproducts, exponentially attenuating metabolic flux to the target molecule as pathway length is increased^9^,^10^,^11^. Consequently, the chemical space we can practically access biosynthetically has been limited to a very small number of steps from natural structures.

Examination of biosynthetic pathways in nature has revealed a common strategy for achieving pathway selectivity: for many natural product pathways, the first committed enzymes serve as highly specific ‘pathway gatekeepers'^12^,^13^. With this type of pathway organization, enzymes further downstream in the pathway need only be moderately selective, since structurally similar but undesirable substrates will have been excluded by the upstream gatekeeper enzyme. Regrettably, engineering highly specific enzymes in the laboratory—in particular, elimination of activity on the native substrate or product—has proven difficult and time-consuming^14^,^15^,^16^,^17^. Accordingly, constructing efficient and selective multi-step pathways to non-natural metabolites is an extremely challenging problem. Approaches taken to improve pathway flux toward non-natural pathway products include enzyme overexpression, deletion of competing endogenous enzymes^7^,^10^,^18^, as well as enzyme fusion, co-localization^11^, compartmentalization^19^ or attachment to scaffolds^20^. These strategies can redirect metabolic flux but do not prevent broad-specificity enzymes from creating unwanted branches. Here, we tackled the construction and focusing of flux for a pathway composed of six successive broad-specificity enzymes, in which an additional strategy was required. A novel strategy, which we term ‘metabolic filtering', allowed us to implement a synthetic pathway to C_50_-astaxanthin, a non-natural, purple analogue of the carotenoid astaxanthin.

Carotenoid biosynthesis has provided an excellent model system for studying the biosynthetic discovery of new chemical structures^12^,^21^,^22^. The pigmentation of carotenoids facilitates screening for altered enzyme function and forms the basis for their nutritional and technological value. All >750 naturally occurring carotenoids are biosynthesized through the modification of C_40_ or C_30_ backbones. While the carotenoid backbone synthase CrtB or CrtM serves as the specific gatekeeper in bacterial C_40_ or C_30_ carotenoid pathways^23^, downstream enzymes are ‘locally specific^12^,^21^', thereby allowing for the creation of non-natural C_30_ or C_40_ carotenoids by mixing and matching (‘combinatorial biosynthesis')^22^,^24^ and sometimes mutating^21^ the enzymes therein.

Previously, chemists synthesized 50-carbon analogues of β-carotene^25^ and astaxanthin^26^, C_50_-β-carotene (**9**) and C_50_-astaxanthin (**12**; also called decapreno-β-carotene^25^ or decaprenoastaxanthin^26^; see Supplementary Note 1 for nomenclature rules). Compared with their natural C_40_ counterparts, the C_50_ carotenoids possessed novel purple (versus orange-to-red) pigmentation^25^ and superior antioxidant activity^27^. Given astaxanthin's (**6**) importance (2010 sales of US$ 226 million^28^) and extended biosynthetic pathway, we settled on C_50_-astaxanthin (**12**) as a target non-natural purple carotenoid product for this study. Almost a decade ago, some of us successfully constructed a non-natural pathway to C_50_-lycopene (**8**) in *E. coli* by engineering carotenoid enzymes^9^,^29^. However, the predominance of smaller carotenoid backbones and the low activity of carotenoid desaturase on the C_50_ backbone (**7**) limited production of C_50_-lycopene (**8**) to only trace quantities (∼a few % of total carotenoids). To further extend the C_50_ pathway to C_50_-astaxanthin (**12**), it would be necessary to dramatically improve the selectivity of the nascent synthetic C_50_ pathway.

Herein, we describe our systematic efforts to rapidly establish a pathway that selectively produces C_50_-astaxanthin (**12**) in *E. coli*. This time, we generated a collection of variants of the first two pathway enzymes, farnesyl diphosphate synthase (FDS) and carotenoid backbone synthase (CrtM), with slightly shifted size-range selectivities to identify the best pairings for the much more selective production of C_50_ backbone (**7**), via the ‘metabolic filtering' effect (detailed below). This selective pathway to the C_50_ backbone allowed us to further evolve the downstream carotenoid desaturase CrtI for improved activity on C_50_ backbone. Subsequently, we co-expressed three broad specificity wild-type carotenoid modification enzymes, resulting in a six-enzyme pathway to C_50_-astaxanthin (**12**) comprising 15 chemical transformation steps. Our synthetic pathway features minimal production of non-target carotenoid byproducts despite containing not a single enzyme with absolute specificity for its primary substrate or product.

## Results

### Design of a pathway to C_50_-astaxanthin

**Figure 1:**
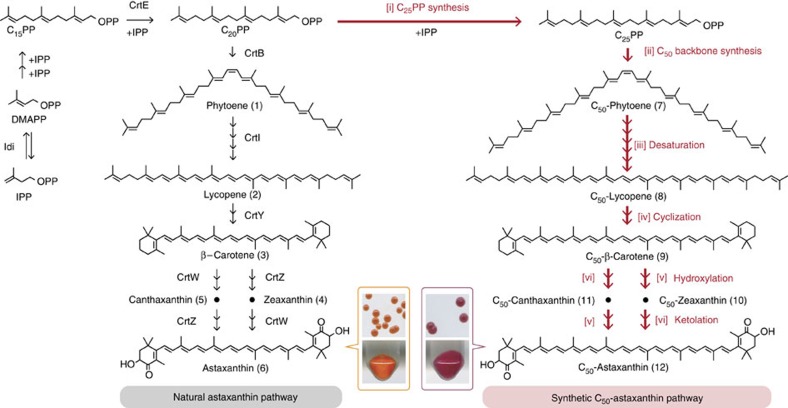
Design of a C_50_-astaxanthin biosynthetic pathway in *E. coli*. Non-natural steps are indicated with red arrows. Starting from endogenous C_15_PP, the pathway includes 15 biochemical steps catalysed by six enzymes (see Supplementary Fig. 1 to see all pathway intermediates). Bottom insets: colonies and cell pellets of *E. coli* expressing pathways to astaxanthin (left) and C_50_-astaxanthin (right) constructed in this study. DMAPP, dimethylallyl diphosphate; IPP, isopentenyl diphosphate; OPP, diphosphate unit.

To introduce a pathway to natural (C_40_) astaxanthin (**6**) (Fig. 1) into *E. coli*^30^, geranylgeranyl diphosphate (C_20_PP) synthase (CrtE) must be expressed to convert endogenous farnesyl diphosphate (C_15_PP) into C_20_PP, the precursor for C_40_ carotenoid synthesis. Phytoene synthase (CrtB) catalyses the head-to-head condensation of two C_20_PPs to phytoene (**1**), the C_40_ carotenoid backbone. Phytoene desaturase (CrtI) extends the double bond conjugation of C_40_ backbone, yielding lycopene (**2**). Terminal β-cyclization (catalysed by CrtY), hydroxylation (by CrtZ) and ketolation (by CrtW) then convert lycopene (**2**) into astaxanthin (**6**). We designed a heterologous biosynthetic route to C_50_-astaxanthin (**12**) by mimicking the astaxanthin (**6**) pathway (Fig. 1). Beginning with endogenous C_15_PP, the pathway proceeds through (i) a two-step prenyl elongation to create geranylfarnesyl diphosphate (C_25_PP); (ii) head-to-head condensation of two C_25_PP to form the C_50_ backbone (**7**); (iii) six desaturation steps to generate the conjugated chromophore; and terminal (iv) β-cyclization; (v) hydroxylation; and (vi) ketolation (Fig. 1, Supplementary Fig. 1 and Supplementary Note 1).

As with most secondary-metabolic enzymes, carotenoid-modifying enzymes, including those for β-cyclization, hydroxylation and ketolation (iv, v, vi), are known to have broad substrate specificity and bind to (or chemically modify) only a portion of their substrate (for example, the terminus)^31^,^32^,^33^. Also, one of us found that *Pantoea ananatis* CrtI (iii) possessed weak but detectable desaturation activity on the C_50_ backbone^9^ (**7**). In contrast, the two upstream enzymes responsible for carotenoid backbone synthesis, isoprenyl diphosphate synthase (i) and carotenoid backbone synthase (ii), are much more selective for their native product and substrate, respectively. However, through enzyme engineering, a broadened-specificity mutant of *Geobacillus stearothermophilus* farnesyl diphosphate (C_15_PP) synthase (FDS) capable of C_25_PP-synthesis, FDS_Y81A_ (i) was generated^34^, as was a set of broadened-specificity mutants of CrtM (a C_30_ carotenoid backbone synthase from *Staphylococcus aureus*) capable of C_50_ backbone synthesis from C_25_PP, including CrtM_F26A,W38A_^29^ (ii).

**Figure 2:**
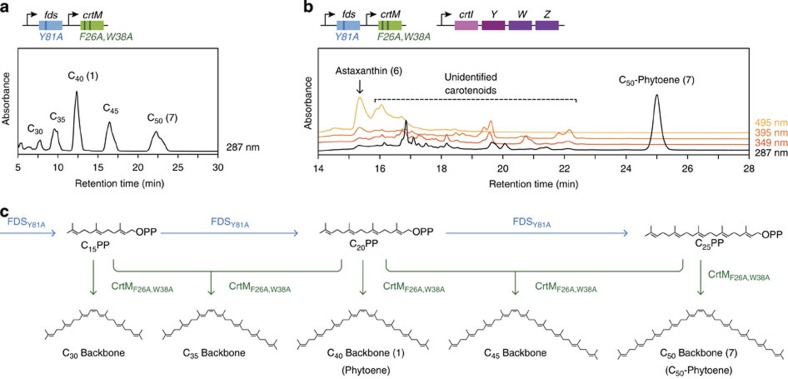
Co-expression of broad-specificity enzymes results in the complex mixture of undesired carotenoid byproducts. (**a**) HPLC trace showing the backbone distribution resulting from co-expression of two enzyme variants, FDS_Y81A_ and CrtM_F26A,W38A_, previously reported to have C_25_PP synthase activity and C_50_ backbone synthase activity, respectively. See **c** for the pathways generated by these enzymes. (**b**) Co-expression of the six broad-specificity enzymes (FDS_Y81A_, CrtM_F26A,W38A_, CrtI, CrtY, CrtW, CrtZ) encoding all of the biochemical functions required in theory to synthesize C_50_-astaxanthin results in a complex mixture of undesired carotenoids. (**c**) Biosynthetic pathways to natural (C_30_, C_40_) and non-natural (C_35_, C_45_ and C_50_) carotenoid backbones by FDS_Y81A_ and CrtM_F26A,W38A_.

Unfortunately, simple co-expression of the above genes (i, ii, iii, iv, v, vi) in *E. coli* resulted in the accumulation of a slew of non-target (mainly C_40_) carotenoids and C_50_-astaxanthin (**12**) was neither separated nor conclusively detected (Fig. 2b). Calculations reveal that based on the catalytic functions reported for the six enzymes, at least 642 different carotenoids can theoretically be biosynthesized from their co-expression (Supplementary Note 2, Supplementary Table 1 and Supplementary Fig. 2). This initial failure to synthesize C_50_-astaxanthin demonstrates that merely co-expressing the enzymes encoding all of the biochemical functions required in theory to synthesize a product does not guarantee a functional multi-step pathway in reality. Because the selectivities of the assembled pathway enzymes were not properly coordinated, carbon flux was diverted at key steps of the pathway along one or more undesirable paths. Convinced that enormous time and effort would be required to evolve the appropriate precise specificity for even one key pathway enzyme^14^,^15^,^16^,^17^, we instead attempted to search for FDS and backbone synthase (CrtM) variants that could combine to effect the selective production of C_50_ backbone.

### Directed evolution of FDS for improved C_25_PP supply

Production of the C_50_ carotenoid backbone (**7**) requires the precursor C_25_PP. A handful of natural C_25_PP-producing enzymes have been described in the literature^35^, but they exhibited insufficient activity and selectivity for C_25_PP^36^. Instead, we had been expressing a variant of FDS (FDS_Y81A_^34^) to produce C_25_PP. However, C_20_PP also accumulated and was readily incorporated into C_40_ and C_45_ backbones by engineered CrtM variants^29^ (Fig. 2a,c). These byproducts diverted carbon flux from the C_50_ pathway and outcompeted C_50_ backbone as substrates for the subsequent steps^9^,^29^.

**Figure 3:**
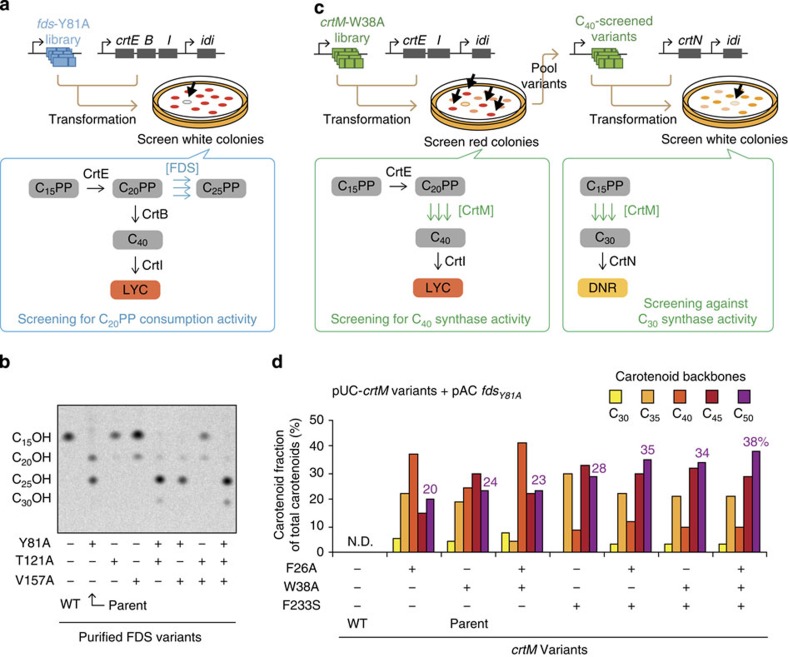
Directed evolution of C_25_PP synthase and C_50_ backbone synthase. (**a**) Directed evolution of a more efficient C_25_PP synthase. The Y81A mutant of FDS was subjected to random mutagenesis and screening for improved C_20_PP consumption. The library was visually assayed in colonies co-expressing *crtE*, *crtB* and *crtI*; hits were colonies with reduced red (lycopene) pigmentation. (**b**) *In vitro* activity of FDS variants provided with DMAPP and [1-^14^C]IPP as substrates. Note that the products have been dephosphorylated to the corresponding alcohol. See Supplementary Note 3 for details. (**c**) Directed evolution of improved C_50_ backbone synthases. Random mutants of CrtM_W38A_ were screened for maintenance of C_40_ synthase function in the presence of *crtE* and *crtI*, followed by selection for reduction of C_30_ synthase function in the presence of *crtN*. (**d**) Effect of selectivity-altering mutations on the cellular activity of CrtM mutants. Carotenoid backbones produced by *E. coli* harbouring pUC-*crtM* mutants and pAC-*fds*_*Y81A*_. FDS_Y81A_ provides C_15_PP, C_20_PP and C_25_PP for the CrtM variants. DNR, diaponeurosporene (C_30_ carotenoid); LYC, lycopene (C_40_ carotenoid).

Lacking a direct high-throughput screen for the production of C_25_PP, we subjected FDS_Y81A_ to random mutagenesis and instead screened the library for improved C_20_PP consumption (Fig. 3a). A library of random point mutants of FDS_Y81A_ was created, and was transformed into *E. coli* cells harbouring a lycopene-producing plasmid (pAC-*crtE-crtB-crtI-idi*), which were then spread on agar plates to form colonies. The rationale was that FDS variants with improved activity for converting C_20_PP into C_25_PP would more fully deplete the lycopene precursor C_20_PP, resulting in whiter colonies. Analysis of the three positive clones identified two additional substrate size-shifting mutations to FDS: T121A and V157A (see Supplementary Note 3, Supplementary Fig. 3 and Supplementary Table 2 for details of the directed evolution and analysis). *In vitro* experiments revealed that subsequent combination of the parental Y81A substitution with either T121A or V157A resulted in shifted selectivity toward larger products (C_25_PP, C_30_PP; Fig. 3b). However, none of these variants was a sole producer of C_25_PP: each produced C_20_PP and/or C_30_PP in addition to C_25_PP, depending on the isoprenyl diphosphate substrates provided (Supplementary Note 3 and Supplementary Fig. 4).

### Directed evolution of CrtM for improved C_50_ activity

Previously, some of the authors had engineered mutants of CrtM (a C_30_ backbone synthase) that could synthesize a C_50_ backbone (**7**), but only as a minor fraction of a complex mixture with C_35_−C_45_ carotenoid backbones^9^,^29^ (Fig. 2a,c). We desired a better C_50_ backbone (**7**) synthase. The lack of a visual or colorimetric screen for identification of variants with improved activity for C_50_ backbone synthesis motivated us to conduct an indirect search for such variants: we searched for CrtM variants with approximately undiminished *in vivo* C_40_ synthase activity but reduced C_30_ synthase activity, both of which could be assayed by colony colour (Fig. 3c). Our hypothesis was that some of the resultant variants would possess an overall shifted preference for larger substrates (Supplementary Note 4 and Supplementary Fig. 5a).

We chose CrtM_W38A_, a broad-specificity variant that catalyses both C_30_ (C_15_PP+C_15_PP) and C_40_ (C_20_PP+C_20_PP) backbone synthesis^29^, as the parent for this evolution experiment (see Supplementary Note 4 for rationale). A library of genes encoding variants of CrtM_W38A_ was co-transformed with pAC-*crtE-crtI-idi* (see left panel in Fig. 3c) into *E. coli* cells and screened for maintenance of C_40_ activity for five rounds. Next, the resultant plasmid mixture was screened for diminished C_30_ synthase activity (right panel in Fig. 3c). Three mutants conferring the desired white phenotype were isolated. Analysis identified F233S as a new size-shifting substitution in CrtM (Supplementary Note 4 and Supplementary Table 3).

When F233S was combined with the previously identified size-altering substitutions^29^ F26A or W38A, the resultant CrtM variants showed increased C_50_ synthesis activity when co-expressed with FDS_Y81A_, while still possessing C_30_−C_45_ synthase activity (Fig. 3d and Supplementary Fig. 5). These variants also acquired unprecedented C_55_ synthase activity when co-expressed with a previously reported C_>30_PP synthase mutant of FDS (Supplementary Fig. 6). This shift in preference for larger substrates was encouraging, but the new CrtM variants remained insufficiently selective for the C_50_ backbone pathway. A strategy for improving their C_50_ selectivity or for otherwise mitigating their undesired backbone synthesis activity was thus required.

### Matching FDS and CrtM variants for C_50_ metabolic filtering

**Figure 4:**
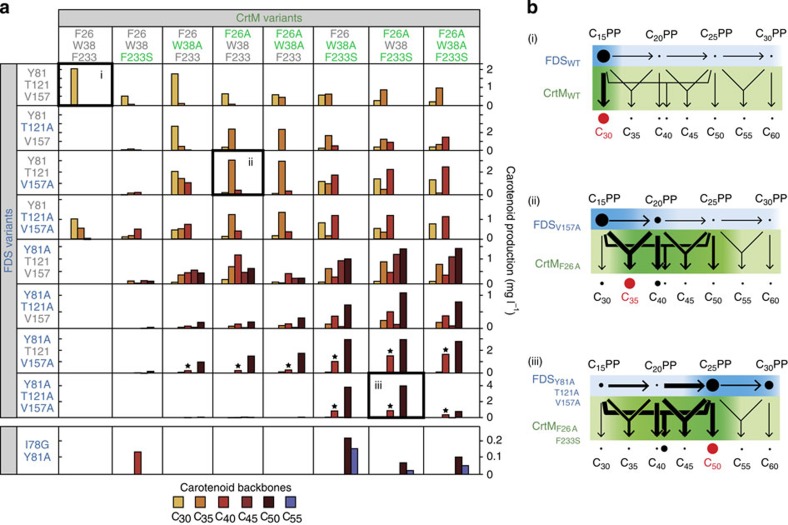
Combinatorial co-expression of enzyme variants for selective production of carotenoid backbones. (**a**) Size distributions of carotenoids produced by combinatorial pairing of FDS and CrtM variants. Bars with stars represent asymmetric C_40_ backbones, produced from C_15_PP+C_25_PP^9^. (**b**) Illustration of metabolic filtering. A modest shift in the precursor distribution effected by the choice of FDS variant, combined with a modest shift in backbone synthase substrate selectivity driven by the choice of CrtM variant can result in substantial focusing of pathway flux to a target product (for example, C_35_ or C_50_ carotenoids). Thicker arrows represent preferred enzyme selectivities, while larger dots represent greater metabolite concentrations. The boxes in **a** labelled i, ii and iii, correspond to the labelled diagrams in **b**. See Supplementary Note 6 and Supplementary Fig. 7 for a more extensive and quantitative illustration.

Next, we investigated all eight combinations of the three FDS mutations (Y81A, T121A, V157A) as well as all eight combinations of the three CrtM mutations (F26A, W38A, F233S) by conducting an 8 × 8 *in vivo* combinatorial expression experiment (Fig. 4) in *E. coli*. The 64 carotenoid distributions were determined by HPLC.

This breeding experiment generated a diverse array of product distributions (Fig. 4a). Although none of the eight FDS or eight CrtM variants possesses stringent substrate or product selectivity, several pairings led to the production of a dominant carotenoid backbone. For example, CrtM_F26A,F233S_ can produce carotenoid backbones from C_30_ to C_55_, depending on the isoprenyl diphosphate synthase with which it is paired (seventh column of Fig. 4a). However, this variant produced C_50_ backbone with high proportion (>80% of total carotenoids) when paired with FDS_Y81A,T121A,V157A_. Similarly, CrtM_F26A_ and CrtM_F26A,W38A_ selectively produced C_35_ backbone when paired with FDS_T121A_ or FDS_V157A_, but yielded various other carotenoid backbones when paired with other FDSs (fourth and fifth column of Fig. 4a). The same holds true for the FDS variants: FDS_T121A_ and FDS_V157A_ selectively produced C_35_ backbone when paired with CrtM_F26A_ or CrtM_F26A,W38A_ but yielded various other carotenoid backbones when paired with other CrtM variants (second and third row of Fig. 4a). Though no FDS variant is itself specific for a particular isoprenyl diphosphate product, a high degree of carotenoid backbone selectivity was achieved with several co-expressed CrtM variants.

**Figure 5:**
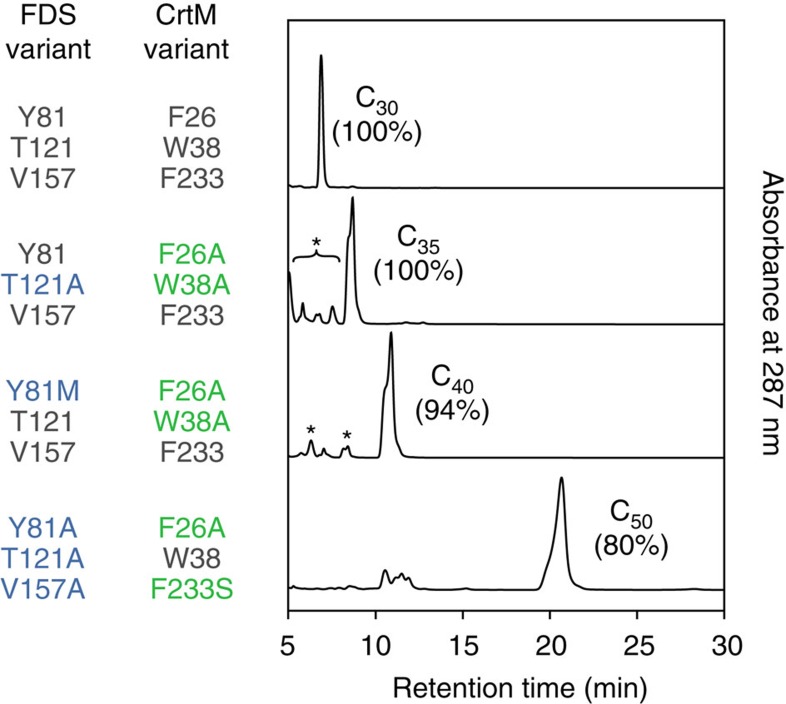
Selective production of C_30_, C_35_, C_40_ and C_50_ backbones by selected combinations of FDS and CrtM variants. Peaks labelled with asterisks correspond to unidentified non-carotenoid compounds. Percentages refer to the mole fraction of total carotenoid backbones.

Inspection of Fig. 4a and multivariate regressions of the underlying data (Supplementary Note 5 and Supplementary Table 4) indicate that the selective production of carotenoid backbones arose from the choice pairing of moderately selective FDS and CrtM enzyme variants, not from exquisite selectivity of any individual enzyme. This contrasts with the dominant contribution of a single specific gatekeeper enzyme, CrtB, to the high production selectivity of C_40_ backbone seen in natural bacterial carotenoid pathways^12^. We term this coordination of successive broad-specificity pathway enzymes to create an appropriate ‘overlap' or ‘channel' between the product range of the first enzyme and the substrate range of the second, ‘metabolic filtering.' We believe metabolic filtering is a rapid and experimentally tractable approach to the yet-undemonstrated alternative of engineering a highly specific single gatekeeper enzyme for focusing pathway flux to a novel product (Fig. 4b, Supplementary Note 6 and Supplementary Fig. 7 provide additional explanations of this concept). We were able to use metabolic filtering to selectively produce carotenoids of various sizes (Fig. 5).

### Directed evolution of CrtI for improved C_50_ activity

**Figure 6:**
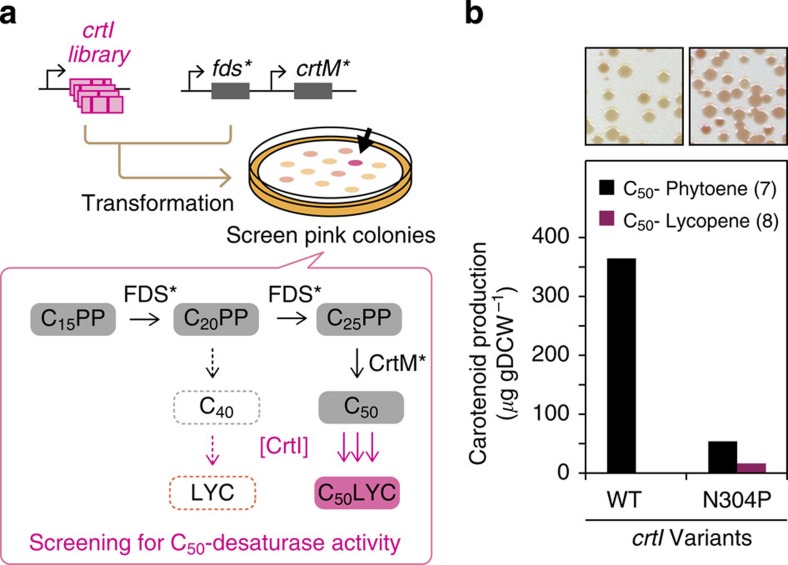
Directed evolution of a C_50_ carotenoid desaturase. (**a**) CrtI was subjected to PCR mutagenesis and colonies were screened for increased pigmentation in cells predominantly producing the C_50_ backbone. Abolition of phytoene was prerequisite for assaying desired C_50_ (versus undesired C_40_) function. (**b**) C_50_ desaturase activity of CrtI and CrtI_N304P_ in *E. coli* expressing FDS_Y81A,V157A_ and CrtM_F26A,W38A,F233S_. Insets show the colony hue of each transformant. CrtM*, CrtM_F26A,W38A,F233S_; FDS*, FDS_Y81A,V157A_.

Previously, *P. ananatis* CrtI was shown to have low but detectable C_50_ backbone (**7**) desaturation activity^9^. Predictably, addition of CrtI to a C_50_-selective pathway yielded only a trace amount of C_50_-lycopene (**8**, Fig. 6b). A highly selective C_50_ backbone pathway was a prerequisite for a simple visual screen of CrtI for improved *in vivo* C_50_ desaturase activity that would not be confounded with improved or altered activity on C_40_ backbone (**1**), its native substrate (Fig. 6a and Supplementary Fig. 8a).

We created a library of genes encoding CrtI variants and transformed them into *E. coli* cells harbouring one of the FDS-CrtM variant pairs that produces C_50_ backbone with high selectivity (pAC-*fds*_*Y81A,V157A*_*-crtM*_*F26A,W38A,F233S*_; Fig. 6a). Out of ∼2,000 colonies surveyed, we isolated 6 with an intense red hue. Sequencing, followed by site-saturation mutagenesis identified top-performing variant CrtI_N304P_ as an efficient six-step C_50_ desaturase (Supplementary Note 7 and Supplementary Table 5). Expression of this variant in the context of a C_50_-selective pathway resulted in ∼90% conversion of C_50_ backbones and some accumulation of the six-step desaturation product C_50_-lycopene (**8**, Fig. 6b). Interestingly, we observed far less C_50_-lycopene (**8**) than expected given the consumption of C_50_ backbone (**7**) and the accumulation level of C_50_-β-carotene (**9**) when a carotenoid cyclase was co-expressed (see below). We therefore attribute the low observed level of C_50_-lycopene (**8**) to instability, rapid decomposition or modification of the molecule. All of the improved C_50_ desaturases we isolated retained uncompromised C_40_ desaturase activity (Supplementary Fig. 8) and are therefore broadened-specificity enzymes, although CrtI_N304P_ did not appear to desaturate asymmetric C_40_ (C_15_PP+C_25_PP) carotenoid backbones. Because we had discovered FDS-CrtM pairings that yielded C_50_ backbones with high selectivity, co-expression of C_50_-enabled broadened-specificity CrtI variants with such pairings resulted in the highly selective accumulation of C_50_ pigments without detectable accumulation of non-C_50_ carotenoids.

### Construction of a selective C_50_-astaxanthin pathway

**Figure 7:**
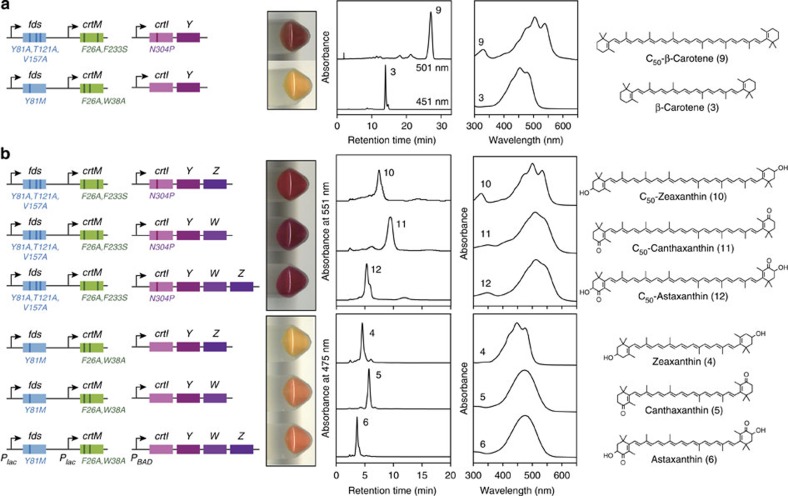
Selective formation of cyclic C_50_ carotenoids. *E. coli* harbouring FDS-CrtM pairs for selective production of C_50_ (FDS_Y81A,T121A,V157A_ and CrtM_F26A,F233S_) or C_40_ (FDS_Y81M_ and CrtM_F26A,W38A_) backbones were additionally transformed with the indicated genes to produce (**a**) C_50_-β-carotene or C_40_ (natural) β-carotene, (**b**) C_50_ oxo-cyclic carotenoids or C_40_ (natural) counterparts. Shown from left to right are the pathway constructs, cell pellets, HPLC chromatograms, absorption spectra and the corresponding carotenoid structure. As illustrated, the plasmid constructs and expression contexts were identical for all wild-type and variants of each gene under comparison (see Supplementary Table 10).

We added downstream modifying enzymes to the resultant pathway for C_50_-lycopene (**8**). This molecule proved a substrate for the wild-type, broad-specificity lycopene cyclase (CrtY) from *P. ananatis*^31^,^37^, such that co-expression of CrtY resulted in the accumulation of C_50_-β-carotene (**9**), essentially as the sole carotenoid product (Fig. 7a). β-Rings are known to photochemically stabilize carotenoid chromophores^27^, and this may explain why the level of C_50_-β-carotene (**9**) was much higher than observed above for C_50_-lycopene (**8**) (Fig. 6b). Further addition of wild-type β-carotene ketolase CrtW and/or hydroxylase CrtZ from *Brevundimonas* sp. SD212, both known to be rather unspecific C_40_ carotenoid enzymes^32^,^33^, yielded the selective production of C_50_-zeaxanthin (**10**), C_50_-canthaxanthin (**11**) and C_50_-astaxanthin (**12**) (Fig. 7b). Thus, we readily extended the C_50_-lycopene (**8**) pathway to a novel C_50_-astaxanthin (**12**) pathway by simple co-expression of three broad specificity wild-type downstream enzymes. C_50_-astaxanthin represented 90% of total carotenoids in the acetone extract; the other 10% was C_50_ backbone. No carotenoids with other backbones were detected. In a further application of metabolic filtering, selective pathways for the natural C_40_ counterpart carotenoids β-carotene (**3**), zeaxanthin (**4**), canthaxanthin (**5**) and astaxanthin (**6**) were also constructed (Fig. 7) simply by matching FDS_Y81M_, the most C_20_PP-selective FDS variant in the published report^34^, with CrtM_F26A,W38A_ one of many proficient C_40_ synthase variants of CrtM.

Without any special metabolic engineering effort to increase carotenoid titre our C_50_-astaxanthin pathway reached a net titre of 920 μg gDCW^−1^ (Supplementary Fig. 9), a value that compares favourably with many titres reported for various natural (C_40_) carotenoids produced in *E. coli* (Supplementary Table 6). Furthermore, this level of membrane-soluble intracellular product was achieved from cells cultured in multiwell plates without control of pH, aeration and other parameters available in a fermenter.

Due to their extended conjugated systems of 15–17 double bonds, the C_50_ carotenoids exhibit substantially red-shifted absorption spectra compared with their respective C_40_ counterparts (Fig. 7 and Supplementary Fig. 10) and are deep-purple in colour. These C_50_ carotenoids have technological potential as colourants or potent antioxidants^27^. In addition, now that it can be produced selectively in *E. coli*, the entire C_50_ pathway is poised to serve as a platform backbone for the exploration of hundreds of potentially functional long-chain carotenoids and derivatives.

## Discussion

The promiscuity of many biosynthetic enzymes enables the creation of novel pathways to non-natural metabolites^12^,^38^,^39^. In our case and in others^9^,^10^,^24^, however, the use of broad-specificity enzymes, especially in the early steps of a novel pathway, has led to a low-titre mixture of numerous products. In cases where a heterologous pathway must compete with an endogenous pathway for substrate, or where ‘leakage' of pathway intermediates to or from endogenous enzymes is an issue, solutions such as enzyme overexpression, deletion, fusion, scaffolding or sequestration have shown effectiveness^7^,^10^,^18^,^19^,^20^. However, these approaches cannot help with the problem of promiscuity-driven pathway branching within the heterologous pathway itself, because the undesirable intermediates both originate from and are processed by heterologous pathway enzymes. Alterations to the selectivity of one or more heterologous pathway enzymes are therefore required to improve flux and selectivity for the desired novel metabolite.

A perfectly ‘lossless' synthetic pathway might require a highly specific enzyme at every step, but we expected it would be immensely difficult to convert FDS and CrtM into variants with exquisite selectivity for their preferred activities^14^,^15^,^16^,^17^,^40^. Instead, we coordinated two successive moderately selective pathway enzymes to ‘share' the role of pathway gatekeeper^12^,^13^ by matching them such that the first enzyme's product range ‘overlaps' partially but appropriately with the second enzyme's substrate range (see Supplementary Note 6). In this way, the two enzymes act together as a ‘metabolic filter' to channel flux along the preferred path. Although some off-pathway (non-carotenoid) byproducts may be generated, C_50_ backbone (**7**) titre was greatly improved from an initial state (Fig. 2a) of ∼40 μg gDCW^−1^ to ∼770 μg gDCW^−1^ (Fig. 5). Crucially, the reduction of on-pathway (mainly C_40_ carotenoid) byproducts enabled the directed evolution of a C_50_ carotenoid desaturase (Fig. 6) and subsequent extension of the C_50_ pathway by 12 additional transformations to C_50_-astaxanthin (**12**). Thus, metabolic filtering was the key that brought the C_50_ pathway to prominence and allowed us to elevate C_50_-astaxanthin (**12**) from undetectable (Fig. 2) to a level comparable to that of C_40_ astaxanthin produced in *E. coli* in this and other studies (Supplementary Fig. 9 and Supplementary Table 6).

This work demonstrates that selective multi-enzyme pathways can be constructed through the judicious matching of individually broad-specificity enzymes and proves that exquisitely specific gatekeeper enzymes are not necessary for achieving a highly selective pathway. This is an encouraging result, with the potential to impact the design and implementation of other synthetic metabolic pathways. In the near future, as synthetic biologists conceive and attempt to establish pathways that diverge ever farther from the natural and use ever-increasing numbers of laboratory-evolved enzymes, the problem of undesired pathway branching due to heterologous enzyme promiscuity will become more widespread and the need for solutions such as this one will grow more critical.

We believe our results and analysis provide a framework for understanding how complex and precise biosynthetic networks can be built out of imperfect biosynthetic parts, either by synthetic biologists or natural evolution. Since tremendous evolutionary effort is usually required for an enzyme to acquire stringent substrate or product specificity^14^,^15^,^16^,^17^,^40^, it makes sense that alternative evolutionary routes to pathway selectivity would have been discovered by nature. Recently, Nam *et al.* showed that 37% of all *E. coli* enzymes are promiscuous and that such enzymes are responsible for 65% of the bacterium's known metabolic steps^41^. Nature therefore seems frequently content to mitigate rather than eliminate the catalytic promiscuity of its enzymes. Our work tangibly demonstrates an additional facet of this phenomenon, and, because so few genetic changes (mutation and lateral gene transfer) were required for us to exploit metabolic filtering, it is very likely that nature discovered this strategy long ago. Mitigation rather than elimination of enzyme promiscuity would preserve nature's ability to rapidly discover new metabolites, and could thus provide some organisms with a selective advantage^12^,^42^. Similarly, the ‘patchwork hypothesis' posits that the selective metabolic pathways we observe in nature evolved from ‘leaky' assemblies of broad-specificity enzymes^43^,^44^. Though individual enzyme specialization has generally been accepted as the primary driver of this evolution^16^,^43^,^44^, the ‘distributed specificity' strategy described herein appears to be a more evolutionarily parsimonious and perhaps, more likely mechanism.

## Methods

### Strains and reagents

*E. coli* XL10-Gold {Tet^r^ *Δ*(*mcrA*)183 *Δ*(*mcrCB-hsdSMR-mrr*)173 *endA1 supE44 thi-1 recA1 gyrA96 relA1 lac* Hte [F' *proAB lacI*^*q*^*ZΔ*M15 Tn*10* (Tet^r^) Amy Cam^r^]} (Stratagene. La Jolla, CA) was used for cloning, while XL1-Blue {*recA1 endA1 gyrA96 thi-1 hsdR17 supE44 relA1 lac* [F' *proAB lacI*^*q*^*ZΔ*M15 Tn*10* (Tet^r^)]} (Stratagene) was used for carotenoid production experiments. Cells were grown Luria-Bertani (LB) in Lennox media for cloning or preculture and in Terrific Broth (TB) media for carotenoid production. Carbenicillin (50 μg ml^−1^; Sigma-Aldrich, St Louis, MO) and/or chloramphenicol (30 μg ml^−1^; Nacalai Tesque, Kyoto, Japan) were supplemented where appropriate. For protein overexpression and purification, BL21-AI (Life Technologies, Carlsbad, CA) was used. For induction, 0.2% (w/v) L-arabinose (Nacalai Tesque) and 0.1 mM IPTG (Nacalai Tesque) was added as an inducer.

### Plasmid construction

Genes and plasmids used in this paper are listed in Supplementary Table 7 and Supplementary Table 8, respectively. Plasmid maps are shown in Supplementary Fig. 11. Plasmids with the prefix ‘pUC' are based on the pUC18m vector^21^, which has an *Eco*RI-*Xba*I-*Xho*I-*Apa*I multi-cloning site under a *lac* promoter. Plasmids with the prefix ‘pAC' are based on the pACmod vector^38^. The plasmids for the downstream enzymes (*crtI, crtY, crtW* and *crtZ*) and their derivatives are based on the pUCara vector. This vector was made by replacing the *lac* promoter of pUC18m with *araC*/*araBAD* promoter PCR-amplified from pBADHisA vector (Invitrogen), together with multi-cloning site (*Eco*RI-*Xba*I-*Xho*I-*Apa*I-*Spe*I-*Cla*I-*Hin*dIII) placed immeditely downstream the *araBAD* promoter. The detailed constructions for each plasmid are provided in Supplementary Note 8. Primer sequences are listed in Supplementary Table 9.

### Plasmid combinations in each experiment

Plasmid combinations used in each experiment are listed in Supplementary Table 10. In brief, for directed evolution and analysis of CrtM, FDS and CrtI (Figs 3 and 6, Supplementary Figs 3 and 5d,e), the gene to be mutagenized was in the pUC18m vector, and additional genes co-expressed for screening were in the pACmod vector. For the production of carotenoid backbones (Figs 2a, 3d, 4 and 5, Supplementary Figs 5 and 6), pairs of pAC-*fds* variants and pUC-*crtM* variants were co-transformed into cells. For the production of desaturated and cyclized carotenoids (Figs 2b, 6b and 7, Supplementary Figs 8–10), pAC-*fds*_*variants*_-*crtM*_*variants*_ (the carotenoid backbone plasmids) were co-transformed with pUCara vector containing downstream enzymes (*crtI* variants and optionally *crtY, crtZ, crtW*).

### Culture conditions for carotenoid backbones

Plasmids harbouring *fds* or *crtM* variants gene were transformed into XL1-Blue cells which were then plated on LB-agar plates and incubated at 37 °C for 24 h. Colonies were inoculated into 2 ml of LB medium in culture tubes, which were shaken at 37 °C for 16 h. The cultures were then diluted 100-fold into 40 ml of fresh TB medium in 200 ml flasks, which were shaken at 30 °C (200 r.p.m.) for 48 h.

### Culture conditions for desaturated carotenoids

For the desaturated carotenoids (cells transformed with *fds* variants, *crtM* variants and one or more downstream enzymes such as *crtI* variants, *crtY*, *crtZ* and *crtW*), the procedure was the same as that for carotenoid backbones, except for the culturing time: the cultures were shaken for 36 h, followed by the addition of L-arabinose to a final concentration of 0.2% (w/v) and an additional 36 h of shaking, unless otherwise indicated.

### Carotenoid extraction and HPLC analysis

Thirty millilitres of each cell culture were centrifuged at 3,300*g* at 4 °C for 15 min. The cell pellets were washed with 10 ml of 0.9% (w/v) NaCl, and then repelleted by centrifugation. Carotenoids were extracted by adding 10 ml of acetone followed by vigorous shaking. One millilitre of hexane and 35 ml of 1% (w/v) NaCl were added, the samples were centrifuged at 3,300*g* for 15 min, and the carotenoid-containing hexane phase was collected. The hexane was then evaporated under a stream of N_2_. The extracts were then dissolved in 100 μl of hexane or (1:1) methanol/THF for separation. A 25-μl aliquot of the final extract was analysed by a Shimadzu Prominence HPLC system (Shimadzu, Kyoto, Japan) equipped with a photodiode array detector.

For the analysis of carotenoid backbones (co-expression culture of *fds* and *crtM* variants; Figs 2a, 3d, 4 and 5, Supplementary Figs 5f,g and 6), a Spherisorb ODS 2 column (250 × 4.6 mm, 5 μm particles; Waters, Milford, MA) was used. Isocratic elution with acetonitrile/isopropanol (6:4 v/v, 1 ml min^−1^) was performed.

For desaturated carotenes (co-expression culture of *fds, crtM* and *crtI* variants, and/or *crtY*; Figs 6b and 7a and Supplementary Fig. 8b–d), a Spherisorb ODS 2 column (see above) was used. The mobile phase was acetonitrile/tetrahydrofuran/methanol (30:7:63 v/v, 1.5 ml min^−1^).

For the analysis of xanthophylls (carotenoids with oxygenic groups; co-expression culture of *fds*, *crtM*, *crtI* variants and *crtY*, together with *crtW* and/or *crtZ*), we used two different elution conditions. For Fig. 2a,b, TSKgel-ODS column and the following gradient elution were employed: solvent A (methanol/water 95:5) for 5 min, gradient from 100% solvent A to 100% solvent B (methanol/tetrahydrofuran 7:3) over the next 5 min, then 100% solvent B for an additional 10 min. For Fig. 7b, a μBondapak column (100 × 8 mm, RCM-type, Waters) was used with methanol as the isocratic mobile phase (1 ml min^−1^).

Individual carotenoids were quantified by their peak areas using a calibration curve generated with known amounts of β-carotene, then multiplying by the molar extinction coefficient (*ɛ*) of β-carotene (138,900 M^−1^ cm^−1^ at 450 nm (ref. 45) and dividing by the *ɛ* value for the carotenoid in question (summarized in Supplementary Table 11). Production weights of carotenoids were then normalized to the dry cell weight (DCW) of each culture. The DCW was calculated using an OD_600_-DCW calibration curve.

### High-throughput analysis of carotenoid production

Analysis of carotenoid production using FDS variants (Supplementary Fig. 3a) or CrtM variants (Supplementary Fig. 5d,e) was performed as follows: transformants (harbouring plasmids indicated in Supplementary Table 10) were plated onto LB Lennox agar plates with appropriate antibiotics to form colonies. These colonies were picked and inoculated into 500 μL LB Lennox media in a 96-well deep well plate and cultured at 37 °C, 1,000 r.p.m. for 16 h. An aliquot (40 μL) of these pre-cultures was transferred to 2 mL TB in a 48-well deep well plate and cultured at 30 °C, 1,000 rpm for 48 h. Cells were collected, washed with saline, centrifuged to obtain cell pellets and the supernatants were discarded. After brief vortex, 1 ml acetone was added to each of the cell pellets, immediately followed by vortex for 1 min and then centrifugation. The absorbance spectra (350–650 nm at 5 nm interval) of the resultant extracts were determined using a SpectraMax Plus^384^ (Molecular Devices, CA). The pigmentation level of each culture was determined from the lambda max (470 nm for C_30_ carotenoids and 475 nm for C_40_ carotenoids) of the resultant extract, using the molar absorption coefficients of diaponeurosporene^45^ (147,000 M^−1^ cm^−1^) or lycopene^45^ (185,000 M^−1^ cm^−1^).

### Pigment identification

Identification of each carotenoid was based on its HPLC retention time and absorption spectrum in the mobile phase. After purification, relative molecular masses of the carotenoids were determined by field-desorption mass spectrometry (FD-MS) with an M-2,500 double-focusing gas chromatograph/mass spectrometer equipped with an FD apparatus (Hitachi, Tokyo, Japan). High-resolution molecular masses were determined by fast atom bombardment (FAB) MS with 3-nitrobenzyl alcohol as a matrix using a JMS-700 110A with an FAB apparatus (JEOL, Tokyo, Japan). The ^1^H NMR (500 MHz) and/or ^13^C NMR (125 MHz) spectra of C_50_ carotenoids in CDCl_3_ were measured at room temperature with a UNITY *INOVA*-500 system (Varian, Palo Alto, CA). Circular dichroism (CD) spectra were recorded in ether at room temperature with a JASCO-J 500 spectropolarimeter. For chemical shift assignments of NMR, see Supplementary Fig. 12. Since the carbon number of C_50_ carotenoids is larger than that of natural (C_40_) carotenoids, the IUPAC-IUB numbering rule of C_40_ carotenoids could not be applied to C_50_ carotenoids. We applied a new numbering rule to the C_50_ carotenoids as we indicate in Supplementary Fig. 12.

*C_50_-β-carotene (**9**)*. Peak 9 of the HPLC chromatogram in Fig. 7a was collected and analysed. Ultraviolet/visible (in methanol): *λ*_max_ 467 (shoulder), 501, 534 nm; FD-MS: *m*/*z* 668. The absorption spectrum indicates a bicyclic carotenoid^46^,^47^.

*C_50_-zeaxanthin (**10**)*. Peak 10 of the HPLC chromatogram in Fig. 7b was collected and analysed. ^1^H NMR (500 MHz, CDCl_3_): *δ* 6.65 (4H, dd, *J*=15, 11 Hz, H-15, 15′, 11, 11′), 6.65 (2H, m, H-19, 19′), 6.38 (2H, d, *J*=15 Hz, H-16, 16′), 6.36 (2H, d, *J*=15 Hz, H-12, 12′), 6.28 (2H, broad d, *J*=11 Hz, H-18, 18′), 6.24 (2H, d, *J*=11 Hz, H-14, 14′), 6.15 (2H, d, *J*=16 Hz, H-8, 8′), 6.15 (2H, d, *J*=11 Hz, H-10, 10′), 6.10 (2H, d, *J*=16 Hz, H-7, 7′), 4.00 (2H, m, H-3, 3′), 2.39 (2H, ddd, *J*=17, 5, 1,5 Hz, H-4α, 4′α), 2.05 (2H, dd, *J*=17, 10 Hz, H-4β, 4′β), 1.99 (6H, s, H-25, 25′), 1.98 (6H, s, H-24, 24′), 1.97 (6H, s, H-23, 23′), 1.77 (2H, ddd, *J*=12, 3, 1.5 Hz, H-2α, 2′α), 1.48 (2H, dd, *J*=12, 12 Hz, H-2β, 2′β), 1.08 (6H, s, H-20, 20′, 21′, 21′) (Supplementary Fig. 13); ultraviolet/visible (in methanol): *λ*_max_ 467 (shoulder), 501, 534 nm; HR FAB MS (*m*/*z*): [M]^+^ calculated (calcd.) for C_60_H_68_O_2_, 700.5219; found, 700.5206; FD-MS: *m*/*z* 700; CD nm (Δ*ɛ*) 350 (0), 348 (−7.5), 330 (0), 278 (−10.6), 255 (0).

*C_50_-canthaxanthin (**11**)*. Peak 11 in Fig. 7b had absorption spectra resembling those of C_50_-β-carotene and C_50_-astaxanthin, respectively. ^1^H NMR (500 MHz, CDCl_3_): *δ* 6.65 (2H, dd, *J*=15, 11 Hz, H-11, 11′), 6.64 (2H, dd, *J*=15, 11 Hz, H-15, 15′), 6.65 (2H, m, H-19, 19′), 6.45 (2H, d, *J*=15 Hz, H-12, 12′), 6.42 (2H, d, *J*=15 Hz, H-16, 16′), 6.37 (2H, d, *J*=16 Hz, H-8. 8′), 6.28 (6H, overlapped, H-18, 18′, 14, 14′, 10, 10′), 6.23 (2H, d, *J*=16 Hz, H-7, 7′), 2.51 (4H, t, *J*=6.5 Hz, H-3, 3′), 2.01 (12H, s, H-24, 24′, 23, 23′), 1.99 (6H, s, H-25, 25′), 1.88 (6H, s, H-22, 22′), 1.85 (4H, t, *J*=6.5 Hz, H-2, 2′), 1.20 (12H, s, H-21, 21′, 20, 20′; Supplementary Fig. 14); ultraviolet/visible (in methanol): *λ*_max_ 512 nm; HR FAB MS (*m*/*z*): [M]^+^ calcd. for C_60_H_64_O_2_, 686.4906; found, 696.4913; FD-MS: *m*/*z* 696.

*C_50_-astaxanthin (**12**)*. Peak 12 of the HPLC chromatogram in Fig. 7b was collected and analysed. ^1^H NMR (500 MHz, CDCl_3_): *δ* 6.66 (2H, dd, *J*=15, 11 Hz, H-11, 11′), 6.65 (2H, dd, *J*=15, 11 Hz, H-15, 15′), 6.65 (2H, m, H-19, 19′), 6.45 (2H, d, *J*=15 Hz, H-12, 12′), 6.43 (2H, d, *J*=16 Hz, H-8. 8′), 6.42 (2H, d, *J*=15 Hz, H-16, 16′), 6.30 (2H, broad d, *J*=11 Hz, H-10, 10′), 6.29 (2H, d, *J*=11 Hz, H-14, 14′), 6.29 (4H, broad d, *J*=11 Hz, H-18, 18′), 6.21 (2H, d, *J*=16 Hz, H-7, 7′), 4.32 (2H, dd, *J*=13.5, 6 Hz, H-3,3′), 3.68 (2H, broad s, OH-3, 3′), 2.15 (2H, dd, *J*=13.5, 6 Hz, H-2α, 2′α), 2.01 (6H, s, H-24, 24′), 2.00 (6H, s, H-23, 23′), 1.99 (6H, s, H-25, 25′), 1.95 (6H, s, H-22, 22′), 1.82 (2H, dd, *J*=13.5, 13.5 Hz, H-2β, 2′β), 1.33 (6H, s, H-20, 20′), 1.22 (6H, s, H-21, 21′) (Supplementary Fig. 15); ^13^C NMR (125 MHz, CDCl_3_): *δ* 200.4 (C-4, 4′), 162.3 (C-6, 6′), 142.5 (C-8, 8′), 140.0 (C-12, 12′), 138.8 (C-16, 16′), 136.8 (C-13, 13′), 135.9 (C-17, 17′), 135.3 (C-10, 10′), 134.2 (C-9, 9′), 134.1 (C-18, 18′), 133.5 (C-14, 14′), 130.5 (C-19, 19′), 126.8 (C-5, 5′), 125.1 (C-15, 15′), 124.1 (C-11, 11′), 123.1 (C-7, 7′), 69.2 (C-3, 3′), 45.4 (C-2, 2′), 36.8 (C-1, 1′), 30.8 (C-21, 21′), 26.2 (C-20, 20′), 14.0 (C-22, 22′), 12.9 (C-25, 25′), 12.8 (C-24, 24′), 12.6 (C-23, 23') (Supplementary Fig. 16); ultraviolet/visible (in methanol): *λ*_max_ 512 nm; HR FAB MS (*m*/*z*): [M]^+^ calcd. for C_50_H_64_O_4_, 728.4805; found, 728.4806; CD nm (Δ*ɛ*) 400 (0), 370 (−3.3), 278 (−3.6), 258 (0), 255 (+0.6), 250 (0), 235 (−3.0), 225 (0). By reduction with NaBH_4_, its absorption spectrum was changed to one strongly resembling that of C_50_-β-carotene, and its molecular mass was increased to 732 (FD-MS), indicating the presence of two carbonyl groups. The presence of two hydroxyl groups was demonstrated by chemical diacetylation and di-trimethylsilylation, which were confirmed by FD-MS analysis^48^.

### Protein purification

*E. coli* BL21-AI (Life Technologies, Carlsbad, CA) was transformed with pET-*fds*_variants_. Fresh colonies were picked, pre-cultured for 14 h, and inoculated into 40 ml LB carbenicillin in 200 ml flasks. Each culture was shaken at 37 °C, 200 r.p.m. and induced with 100 μM IPTG and 0.2%(w/v) arabinose when the OD_600_ reached 0.6–0.8 (approximately 1.5 h). Each culture was then shaken for an additional 4 h before harvest.

One millilitre of B-PER reagent (Pierce Biotechnology, Rockford, IL), supplemented with DNase I, lysozyme, 0.1 mM PMSF, 500 mM NaCl, and 25 mM imidazole, was used to lyse each cell pellet. FDS variants were purified from the lysate using a His SpinTrap column and desalted using a PD MiniTrap G-10 column (both from GE Healthcare), following the manufacturer's instructions. The buffers used were: binding buffer (50 mM Tris-HCl pH8.5, 500 mM NaCl, 25 mM imidazole), elution buffer (50 mM Tris-HCl buffer pH8.5, 500 mM NaCl, 500 mM imidazole) and desalting buffer (50 mM Tris-HCl pH8.5). The concentrations of the resultant protein variants were measured by BCA assay (Pierce BCA Protein Assay Kit), and the purified proteins were stored at −20 °C after adding 5 mM DTT and 15% glycerol.

### *In vitro* reaction of FDS variants

The assay was performed as described previously^34^ with slight modifications. Each assay mixture contained, in a final volume of 200 μl, 1 μmol of MgCl_2_, 10 μmol of NH_4_Cl, 10 μmol of 2-mercaptoethanol, 10 μmol of Tris-HCl buffer (pH 8.5), an appropriate amount of purified FDS variant and [1-^14^C]IPP (3 Ci mol^−1^) and DMAPP as substrates (see also Supplementary Fig. 4 for experimental conditions). The enzyme amount was set to a level such that <20% of the IPP or DMAPP would be consumed under the following reaction conditions. The mixture was incubated at 30 °C for 5 min, and the reaction was stopped by adding 400 μl of ice-chilled saturated NaCl solution. The mixture was shaken with 600 μl of 1-butanol that had been saturated with NaCl. The radioactivity in the 1-butanol layer was determined with a liquid scintillation counter (LSC-5,100, Aloka, Japan). The resulting polyprenyl diphosphates in 1-butanol were treated with acid phosphatase at 37 °C overnight according to the method of Fujii *et al.*^49^. The hydrolysates were extracted with *n*-pentane and analysed by reversed-phase thin layer chromatography using a precoated plate, LKC18-F (GE Healthcare, USA) or RP18 (Merck, Germany), developed with acetone/H_2_O (9:1). The radioactivities of the spots were measured with a Typhoon-FLA 7,000 (GE Healthcare, USA).

## Additional information

**How to cite this article:** Furubayashi, M. *et al.* A highly selective biosynthetic pathway to non-natural C_50_ carotenoids assembled from moderately selective enzymes. *Nat. Commun.* 6:7534 doi: 10.1038/ncomms8534 (2015).

## Supplementary Material

Supplementary Information Supplementary Figures 1-16, Supplementary Tables 1-11, Supplementary Notes 1-8 and Supplementary References
