# Supplementary material for: A highly selective biosynthetic pathway to non-natural C50 carotenoids assembled from moderately selective enzymes
Source: Nat Commun. 2015 Jul 14;6:7534. doi: 10.1038/ncomms8534 (PMC4510654; doi:10.1038/ncomms8534)
Supplement: Supplementary Information — Supplementary Figures 1-16, Supplementary Tables 1-11, Supplementary Notes 1-8 and Supplementary References [file ncomms8534-s1.pdf]

Supplementary Figure 1

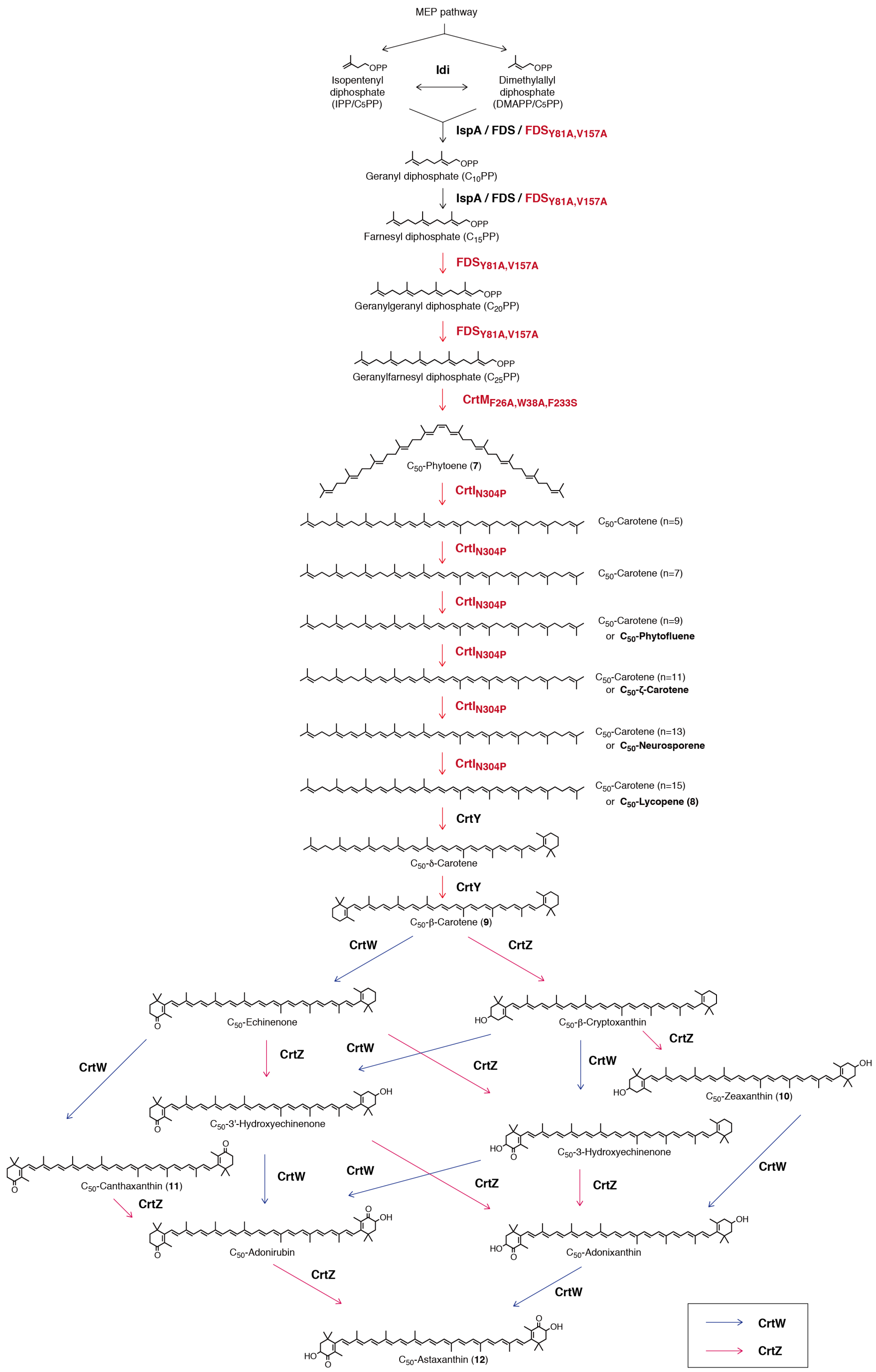

Supplementary Figure 1. Details on the designed pathway toward C_{50}-astaxanthin. Steps of the designed pathway are indicated with colored arrows. Enzyme mutants created in this study ($FDS_{Y81A,V157A}$, $CrtM_{F26A,W38A,F233S}$ and $CrtI_{N304P}$) are indicated in red lettering.

Supplementary Figure 2

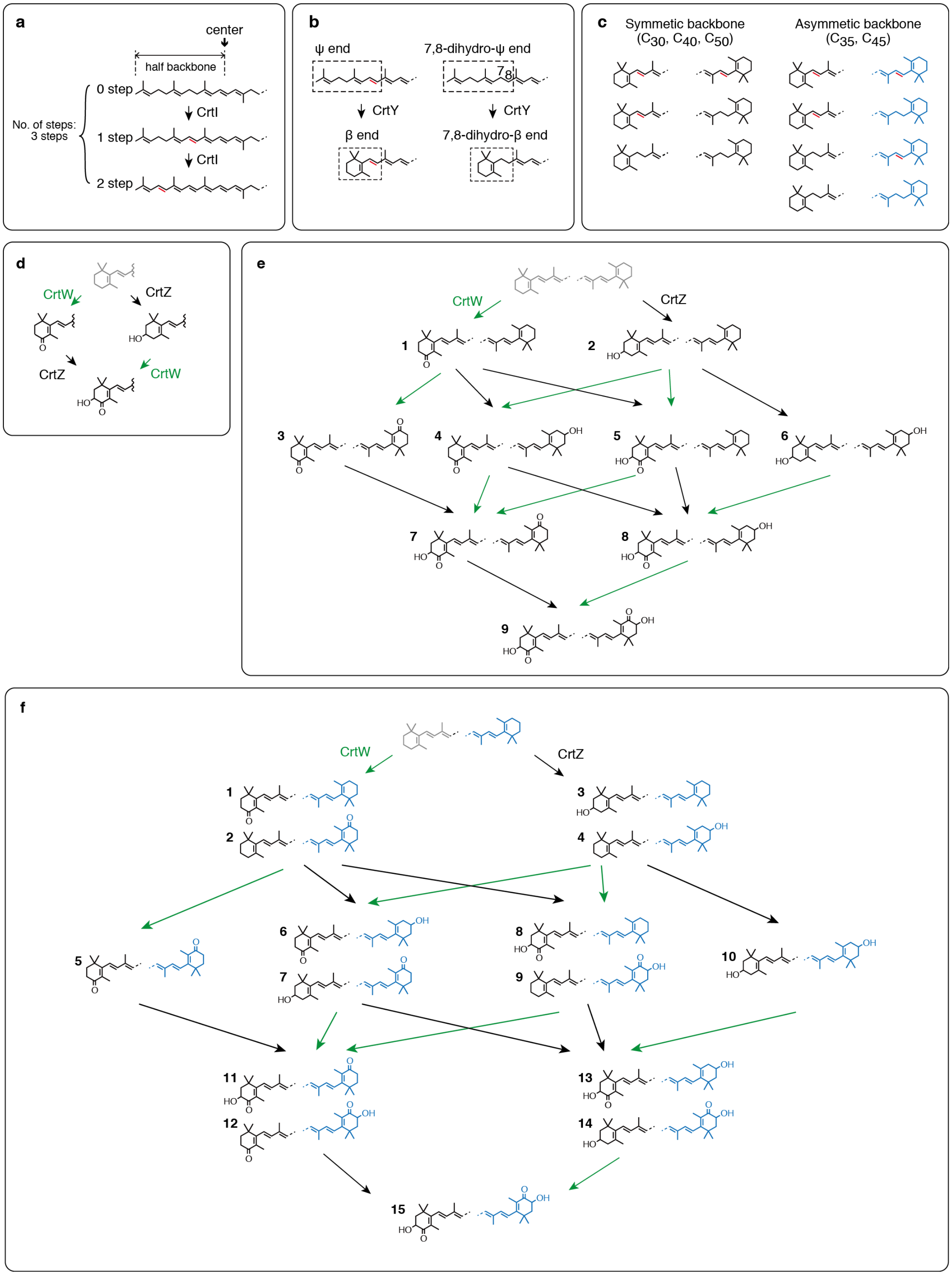

Supplementary Figure 2. The local structure of carotenoids used for calculation in Supplementary Note 2 and Supplementary Table 1.

Supplementary Figure 3

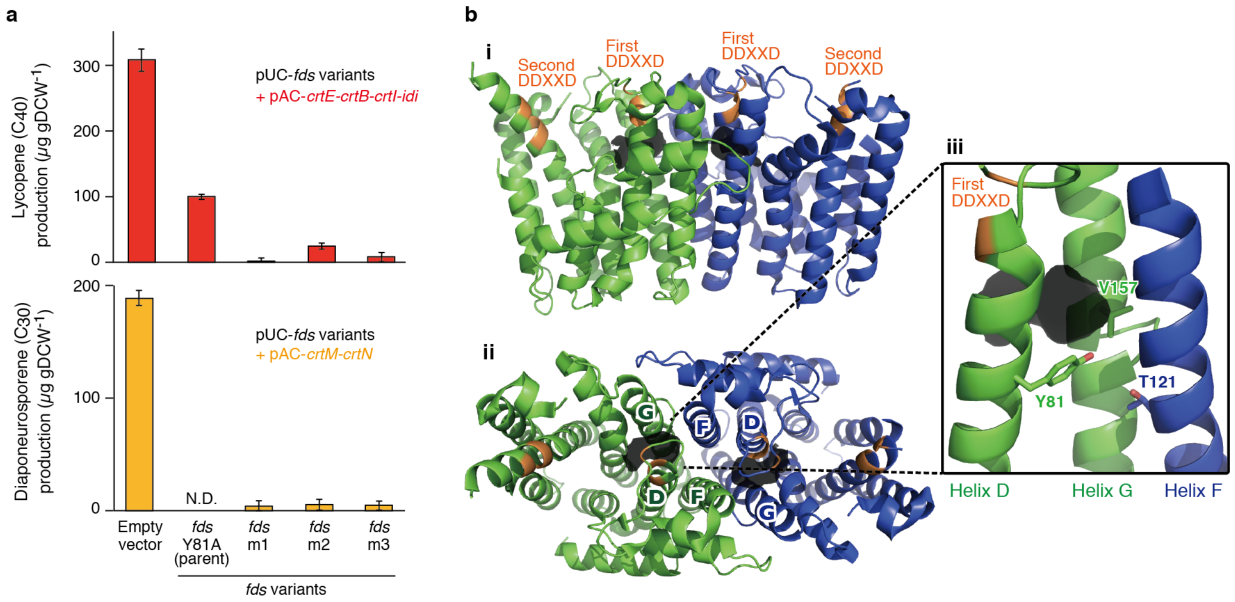

Supplementary Figure 3. Characterization of FDS variants. (**a**) Capacity of FDS variants for C_{15}PP or C_{20}PP consumption *in vivo*. The level of lycopene (C_{40}) or diaponeurosporene (C_{30}) production by *E. coli* cells harboring pAC-*crtE-crtB-crtI-idi* or pAC-*crtM-crtN*, respectively, was measured for cultures expressing different FDS variants on a pUC vector. Bars represent the average of four replicates; error bars represent ± 1 standard deviation. N.D., Not detected. (**b**) Mutations mapped on the structure of *S. aureus* FDS (PDB ID: 1RTR), which shares 46% identity with *G. stearothermophillus* FDS. The substrate cavity (black surface) is located at the dimer interface of FDS, surrounded by helices G, D in one chain, and F from the other. Subpanels (**i**) and (**ii**) provide different views of the same structure. (**iii**) Zoomed view of the substrate cavity. Y81, V157 and T121 are located near the substrate cavity.

Supplementary Figure 4

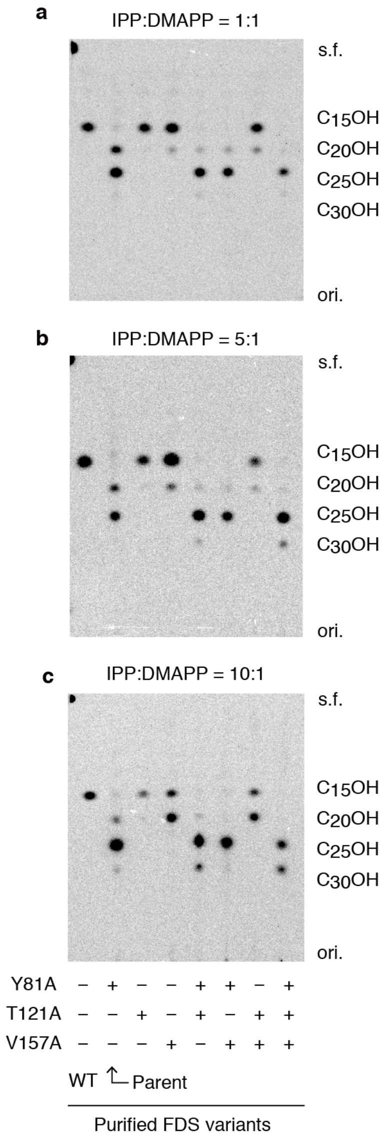

Supplementary Figure 4. *In vitro* analysis of FDS variants. The molar ratio of substrates was (**a**) IPP:DMAPP=1:1 (5 nmol each), (**b**) IPP:DMAPP=5:1 (10 nmol and 2 nmol), or (**c**) IPP:DMAPP=10:1 (16 nmol and 1.6 nmol). The TLC autoradiogram shown in **Fig. 3b** in the main text is the same as panel **b** in this figure. The *in vitro* experiment was performed as described in Methods. Note that the products have been dephosphorylated to the corresponding alcohol. Abbreviations: s.f. solvent front, ori. origin.

Supplementary Figure 5

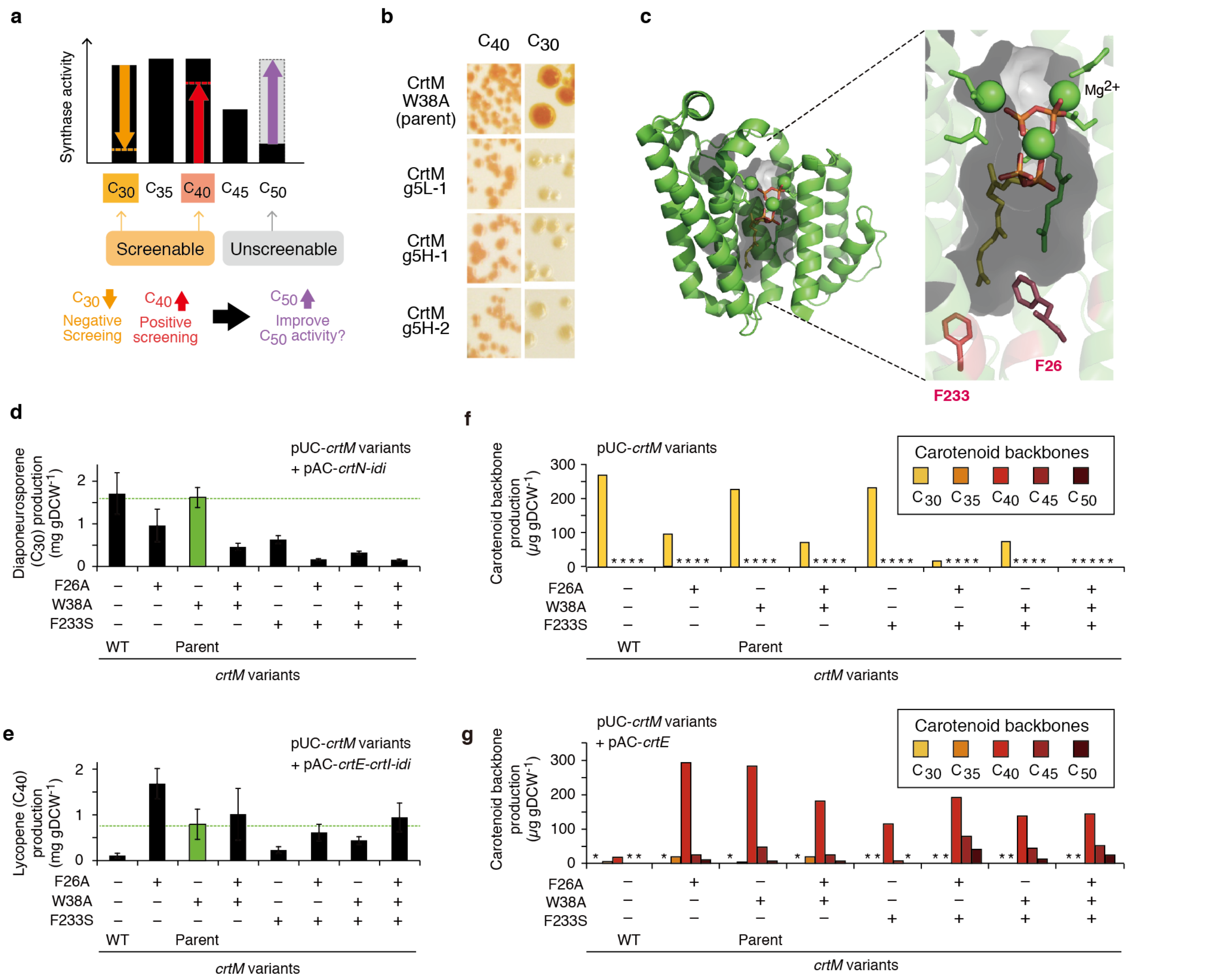

Supplementary Figure 5. Directed evolution of CrtM$_{W38A}$ for the production of larger nonnatural carotenoids. (**a**) We hypothesized that we could shift the specificity range of CrtM toward larger ($C_{>40}$) carotenoids by accumulating mutations neutral to C_{40} function but deleterious to C_{30} function. (**b**) C_{30} and C_{40} synthase functions of isolated CrtM variants. Three mutants and their parent, CrtM$_{W38A}$ (on a pUC vector) were co-expressed with either pAC-*crtN-crtNb-idi* or pAC-*crtE-crtI*. (**c**) Mutation F233 and F26 mapped onto the structure of CrtM (2ZCCP). (**d,e**) The level of diaponeurosporene (C_{30}) or lycopene (C_{40}) production by *E. coli* cells harboring pUC-*crtM* variants, together with pAC-*crtN-idi* or pAC-*crtE-crtI-idi*, respectively. Carotenoid pigments were extracted from the cell pellets and absorbance at 470 nm (d) or 475 nm (e) were used to calculate the carotenoid amounts. Bars represent the average of four replicates; error bars represent ± 1 standard deviation. (**f,g**) Carotenoid backbone production by *E. coli* cells harboring pUC-*crtM* variants (f) or pUC-*crtM* variants and pAC-*crtE* (g). Carotenoids were extracted and analyzed by HPLC (see Methods). Asterisks indicates that carotenoid production was non-detectable.

Supplementary Figure 6

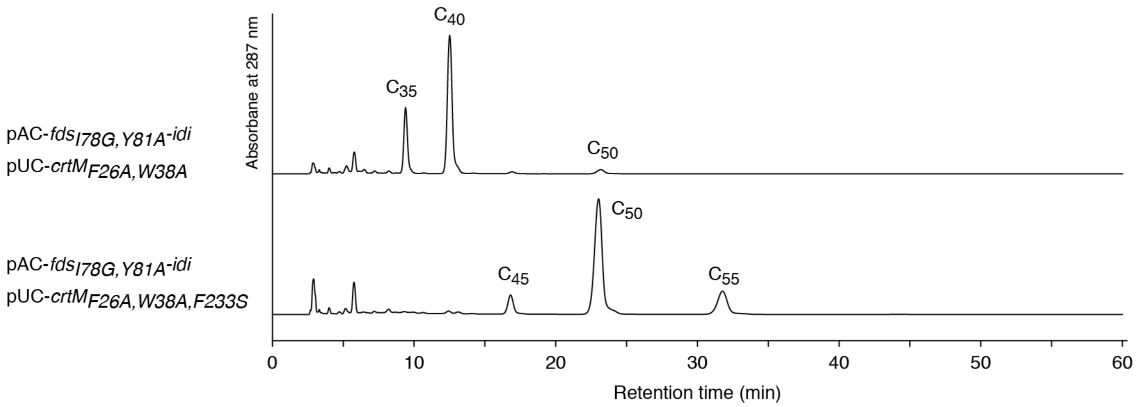

Supplementary Figure 6. The production of C_{55} backbone. Shown are HPLC traces at 287 nm of carotenoid extracts of *E. coli* harboring the indicated plasmids. The identity of the novel large C_{55} backbone was confirmed by mass spectrometry, absorption spectrum (287 nm), and retention time.

Supplementary Figure 7

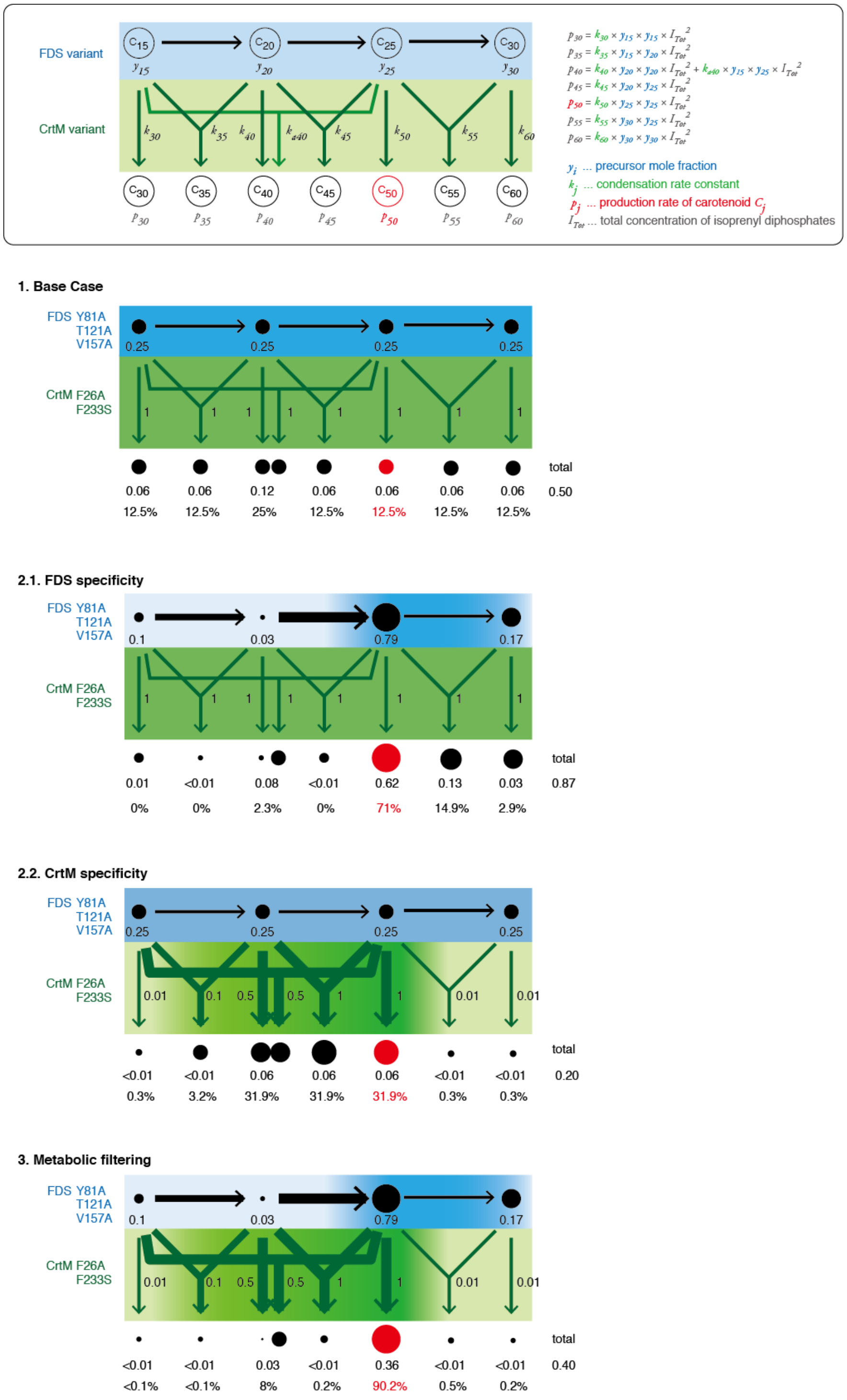

Supplementary Figure 7. Mathematical illustration of metabolic filtering.

Thicker arrows represent preferred enzyme specificities, while larger dots represent greater metabolite concentrations. Numbers beside dots denote relative concentrations; numbers beside arrows denote relative fluxes. For details, see **Supplementary Note 6**.

Supplementary Figure 8

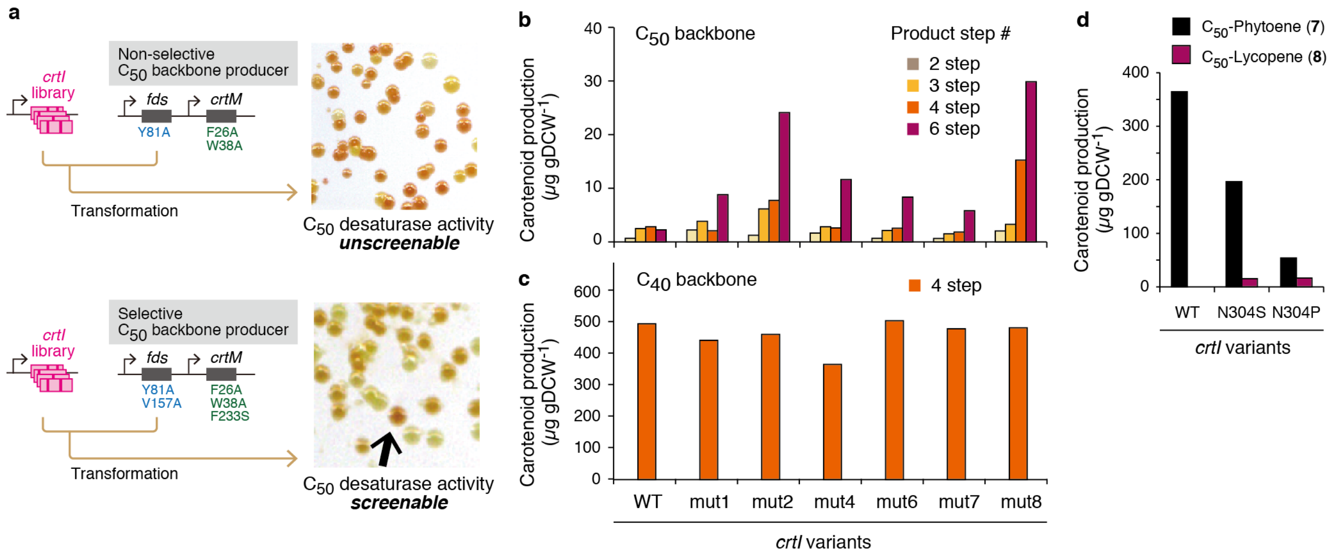

Supplementary Figure 8. Directed evolution of CrtI for C_{50} desaturase activity. (**a**) A specific C_{50} backbone pathway was required to screen for C_{50} desaturase activity. When the FDS_{Y81A}-$CrtM_{F26A,W38A}$ pairing was used to provide carotenoid backbones, there appeared many red colonies upon transformation of the CrtI library (upper panel). This was due to the production of various carotenoid backbones, including undesired phytoene (symmetric C_{40} backbone), the native substrate of CrtI. Colonies were red (false positives for C_{50} desaturation) as long as the desaturase retained C_{40}-desaturation activity. When the $FDS_{Y81A,V157A}$-$CrtM_{F26A,W38A,F233S}$ pairing was used, transformation of the CrtI library resulted in a majority of pale colonies (lower panel), due to the absence of phytoene and poor C_{50} desaturase function. From the rare red clones found in this condition, we isolated CrtI variants with significantly improved C_{50} desaturation activity. (**b**) Pigment analysis of cells expressing CrtI variants and producing the C_{50} backbone. pUCara-*crtI* variants were co-transformed with pAC-$fds_{Y81A,V157A}$-$crtM_{F26A,W38A,F233S}$. The experiments were performed as described in Methods except for a change to the culturing conditions: the inoculated culture was incubated for 20 h, followed by the addition of 0.2% (w/v) L-arabinose and then an additional 18 h of shaking. This condition was used because the normal condition (72 h culture total) yielded excessive degradation of C_{50} desaturated products. (**c**) Pigment analysis of cells harboring CrtI variants with C_{40} backbone. pUCara-*crtI* variants were co-transformed with pAC-fds_{Y81M}-$crtM_{F26A,W38A}$ and analyzed as described in Methods. (**d**) Comparision of wild-type, N304S and N304P variants of *P. ananatis* CrtI for the desaturation of C_{50} carotenoids. pUCara-*crtI* variants were co-expressed with pAC-$fds_{Y81A,V157A}$-$crtM_{F26A,W38A,F233S}$ and analyzed as described in Methods.

Supplementary Figure 9

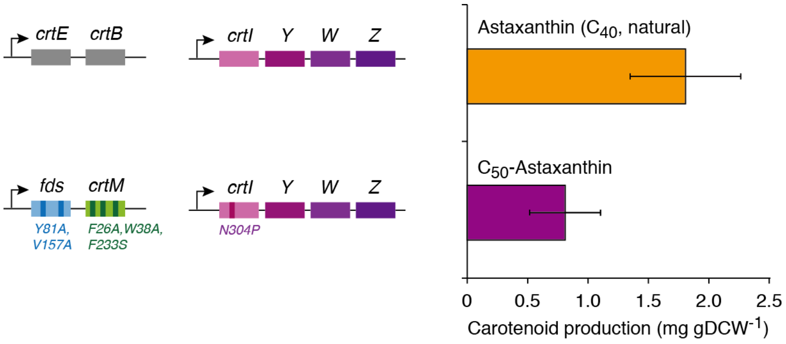

Supplementary Figure 9. Production of astaxanthin and C_{50}-astaxanthin in *E. coli*. Production of carotenoids from cultures of *E. coli* XL1-Blue cells harboring: pAC-$fds_{Y81A,V157A}$-$crtM_{F26A,W38A,F233S}$ and pUCara-$crtI_{N304P}$-*crtY*-*crtW*-*crtZ*. Colonies were inoculated into 2 mL TB medium in 48-deepwell blocks and cultured at 30 °C, 1000 rpm. After 36 h, 0.2%(w/v) arabinose was added to induce downstream enzymes. 36 h after induction, cells were harvested and carotenoids were extracted with acetone. Carotenoid amounts were calculated by measuring absorption at 480 nm (astaxanthin) or 515 nm (C_{50}-astaxanthin), and using the molar extinction coefficient indicated in **Supplementary Table 11**. Bars represent the average of four replicates; error bars represent ± 1 standard deviation.

Supplementary Figure 10

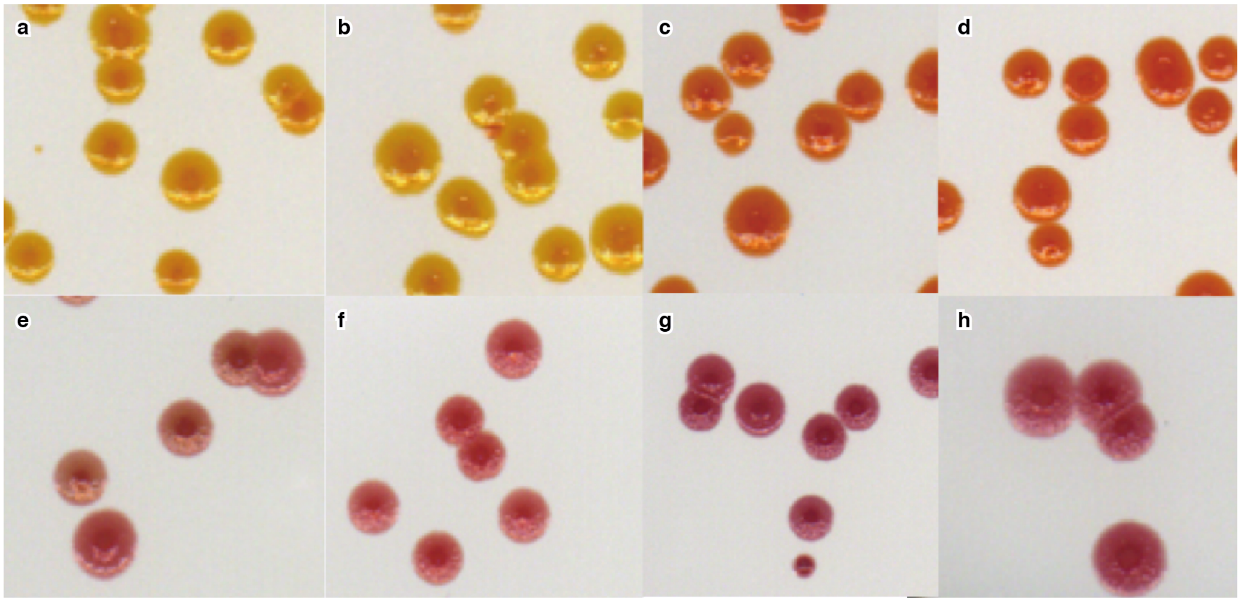

Supplementary Figure 10. The color of *E. coli* colonies harboring specific pathways for (a-d) C_{40} and (e-h) C_{50} carotenoids. For the selective production of C_{40} carotenoids, pAC-fds_{Y81M}-$crtM_{F26A,W38A}$ was co-expressed with (**a**) pUCara-*crtI-crtY* (to produce β-carotene), (**b**), pUCara-*crtI-crtY-crtZ* (zeaxanthin), (**c**) pUCara-*crtI-crtY-crtW* (canthaxanthin), and (**d**) pUCara-*crtI-crtY-crtW-crtZ* (astaxanthin). For the selective production of C_{50} carotenoids, pAC-$fds_{Y81A,V157A}$-$crtM_{F26A,W38A,F233S}$ was co-expressed with (**e**) pUCara-$crtI_{N304P}$-*crtY* (C_{50}-β-carotene), (**f**) pUCara-$crtI_{N304P}$-*crtY-crtZ* (C_{50}-zeaxanthin), (**g**) pUCara-$crtI_{N304P}$-*crtY-crtW* (C_{50}-canthaxanthin), and (**h**) pUCara-$crtI_{N304P}$-*crtY-crtW-crtZ* (C_{50}-astaxanthin). Cells were plated on LB-agar topped with a nitrocellulose membrane to provide a white background.

Supplementary Figure 11

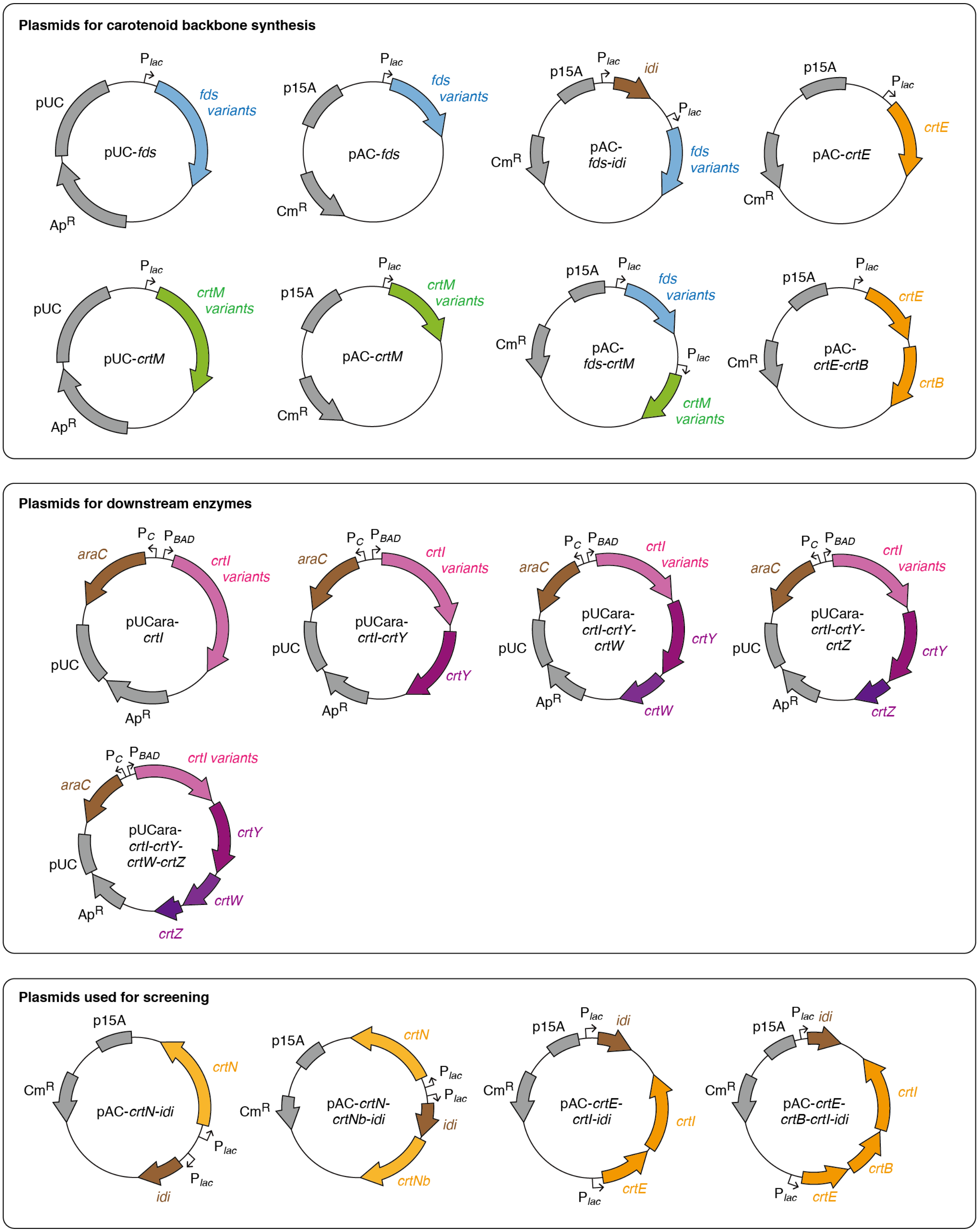

Supplementary Figure 11. Maps of plasmids used in this study.

Supplementary Figure 12

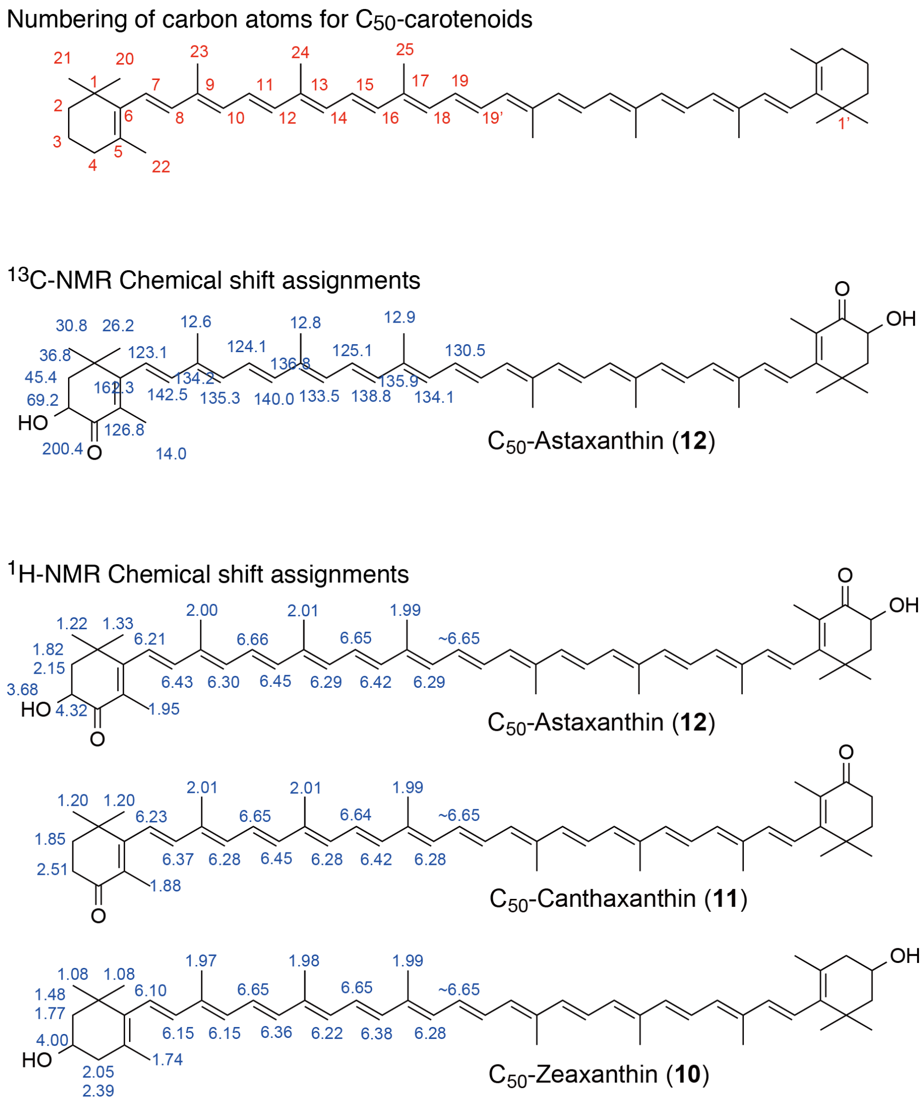

Supplementary Figure 12. 1H-NMR of C_{50}-astaxanthin, C_{50}-canthaxanthin, and C_{50}-zeaxanthin in $CDCl_3$. Carbon numbers of C_{50} carotenoids are indicated in red. Chemical shifts are indicated in blue. Chemical shifts around the β-end group were similar to those of their C_{40} counterparts (lipid bank: http://lipidbank.jp/).

Supplementary Figure 13

^{1}H NMR spectra of C_{50}-zeaxanthin.

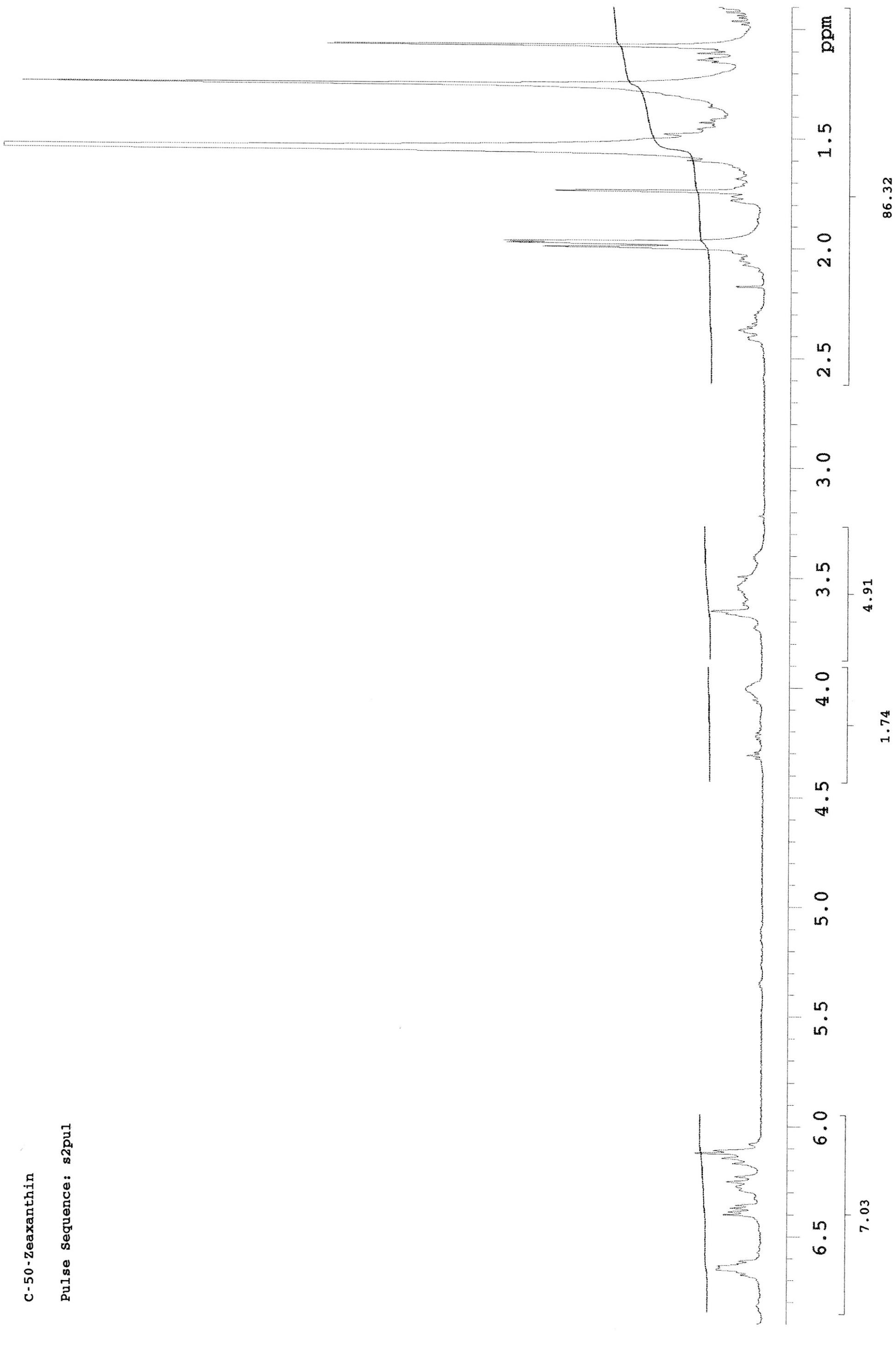

Supplementary Figure 14

^{1}H NMR spectra of C_{50}-canthaxanthin.

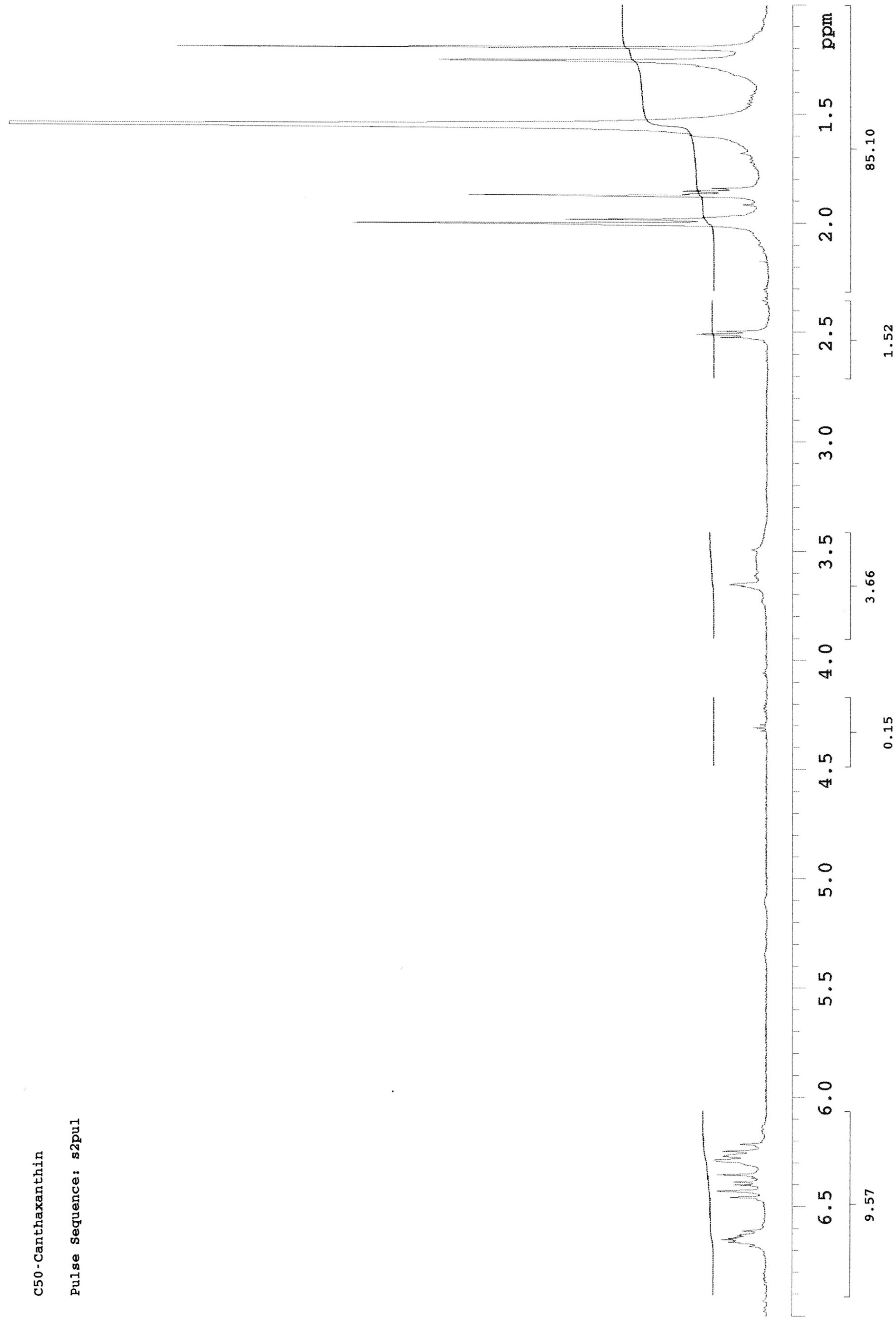

Supplementary Figure 15

^1H NMR spectra of C_{50}-astaxanthin.

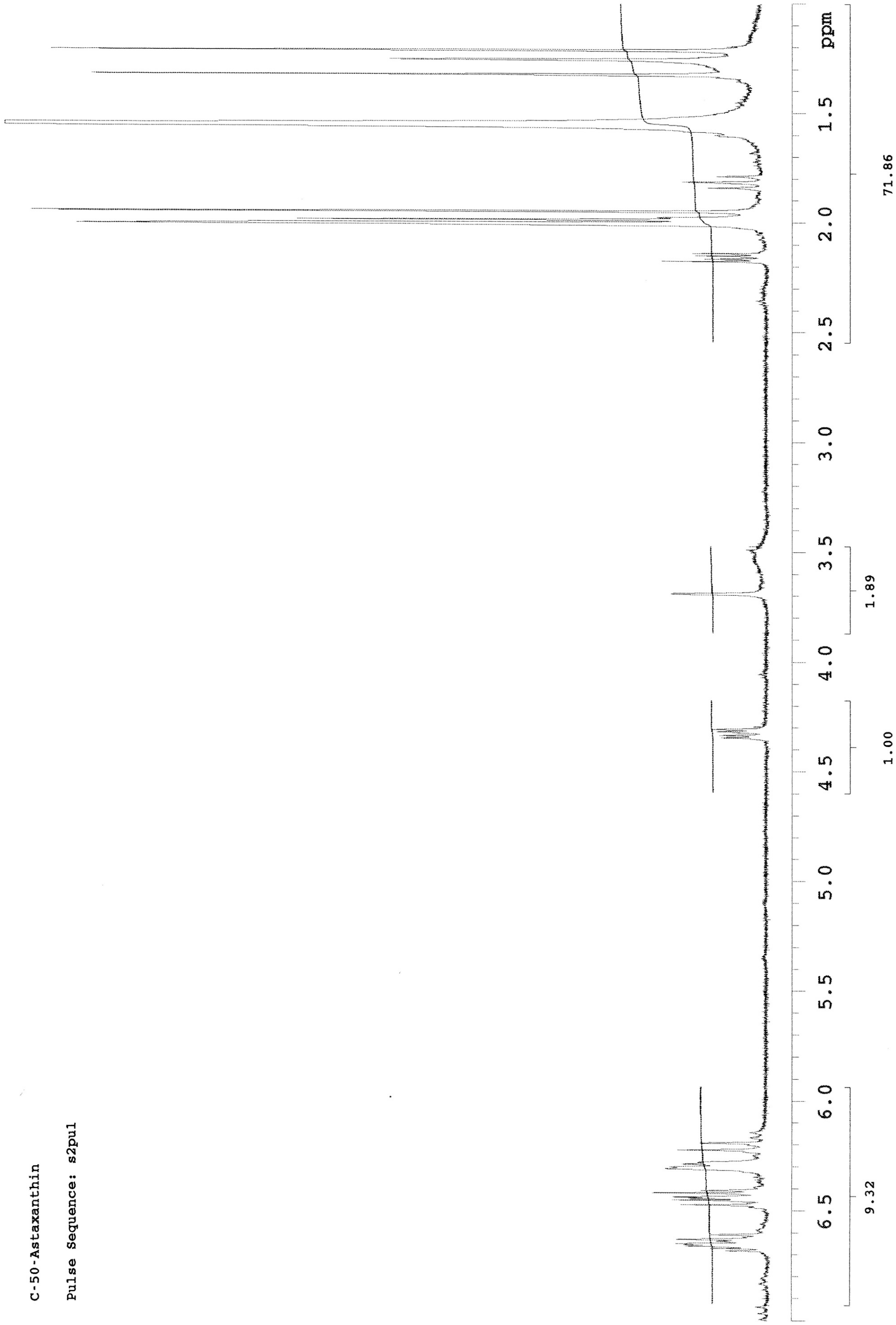

Supplementary Figure 16

^{13}C NMR spectra of C_{50}-astaxanthin.

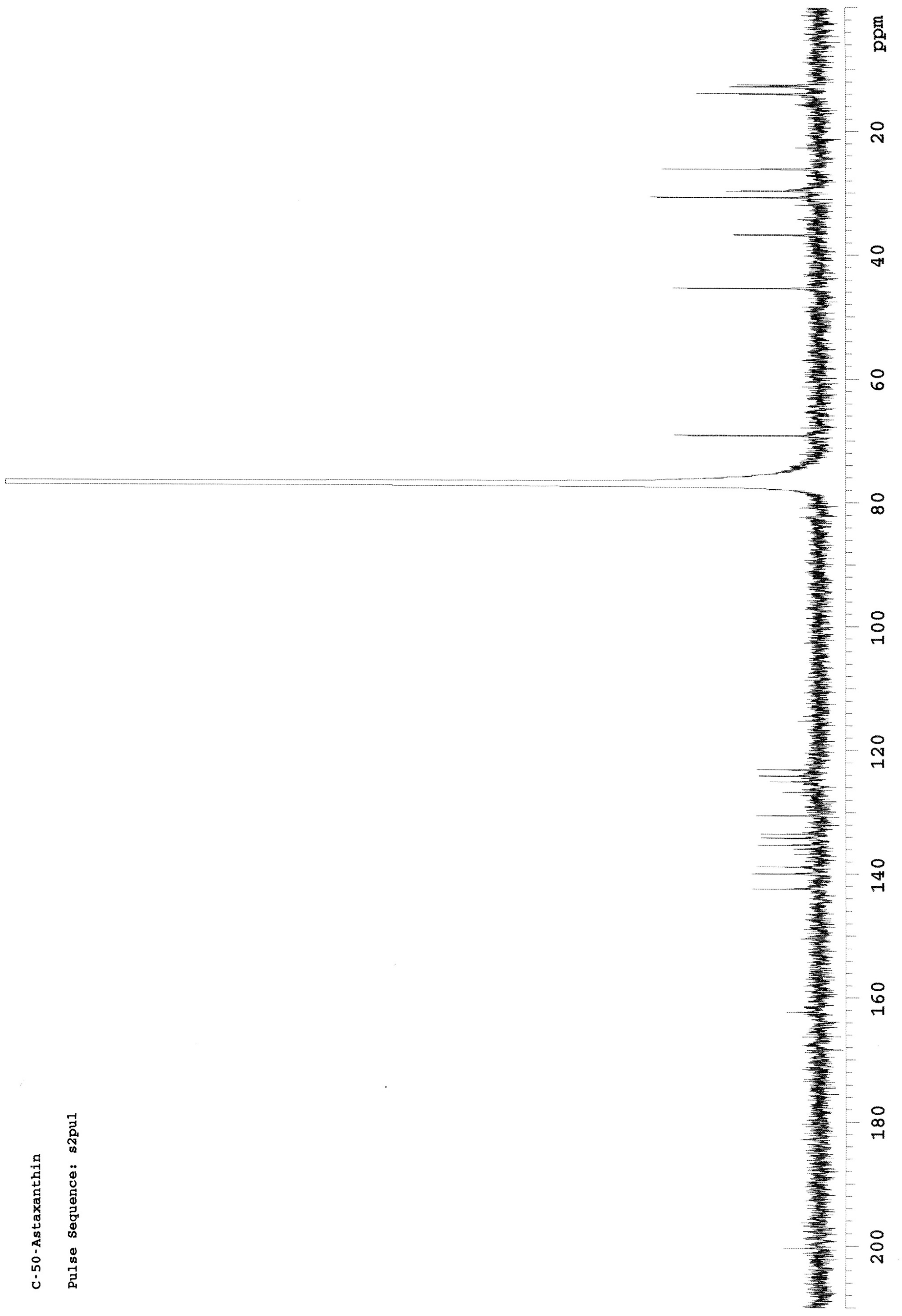

Supplementary Table 1

The potential number of products accumulated by expressing promiscuous carotenoid enzymes. The alphabets, A to E, corresponds to the section in Supplementary Note 2.

Backbone	Desaturation		Cyclization		Oxidation			
	Max. # steps	A. Desaturated Products	B. Singly Cyclized	C. Doubly Cyclized[c]	D. Single cyc/oxy	E. Double cyc/oxy[e]	Subtota l	Total
C_{30} (C_{15}+C_{15})	3 + 3	${}_{3+2-1}C_2$ = 6	3 x 2 = 6	3	6 x 3 = 18	33	66	
C_{35} (C_{15}+C_{20})	3 + 4	3 x 4 = 12	(3+4) x 2 = 14	4	14 x 3 = 42	60	132	
C_{40} (C_{20}+C_{20})	4 + 4	${}_{4+2-1}C_2$ = 10	4 x 2 = 8	3	8 x 3 = 24	33	78	
aC_{40} (C_{15}+C_{25})	3 + 5	3 x 5 = 15	(3+5) x 2 = 16	4	16 x 3 = 24	60	119	
C_{45} (C_{20}+C_{25})	4 + 5	4 x 5 = 20	(4+5) x 2 = 18	4	18 x 3 = 54	60	156	
C_{50} (C_{25}+C_{25})	5 + 5	${}_{5+2-1}C_2$ = 15	5 x 2 = 10	3	10 x 3 = 30	33	91	642
C_{55} (C_{25}+C_{30})	5 + 6	5 x 6 = 30	(5+6) x 2 = 22	4	22 x 3 = 66	60	182	
C_{60} (C_{30}+C_{30})	6 + 6	${}_{6+2-1}C_2$ = 21	6 x 2 = 12	3	12 x 3 = 36	33	105	287
								929

Supplementary Table 2

Genotypes of the FDS variants with improved C_{20}PP consumption.

Variant	Nucleotide and amino acid substitutions in addition to Y81A (nucleotide substitutions not followed by an amino acid substitution in brackets are silent)
FDSm1	A361T (**T121S**)
FDSm2	G96A, A361G (**T121A**), A644G (**H215R**), C715A (**P239T**), T796C (**F266L**)
FDSm3	T470C (**V157A**), T589C, G759T

Supplementary Table 3

Genotypes of the isolated CrtM mutants.

Variant	Nucleotide and amino acid substitutions in addition to W38A (nucleotide substitutions not followed by an amino acid substitution in brackets are silent)
$CrtM_{g5L-1}$	A16G (**M6V**), A35G (**H12R**), T76C (**F26L**), A242G (**Q81R**), T318G (**N106K**), T698C (**F233S**)
$CrtM_{g5L-2}$	A284G (**Q95R**), T414C, G426T, A533T (**D178V**), A602G (**Q201R**), T697C (**F233L**), A814G (**K272E**)
$CrtM_{g5H-1}$	A16G (**M6V**), A35G (**H12R**), T76C (**F26L**), A242G (**Q81R**), A242G (**F233S**)

Supplementary Table 4.

Multiple regression analysis of the carotenoid backbone titer data behind Fig. 4a. See Supplementary Note 5 for discussion.

		C_{30}	C_{35}	C_{40}	C_{45}	C_{50}
		Regression R^2 values				
		0.685	0.630	0.771	0.692	0.704
Model Terms		*p*-values for Model Terms				
FDS	Y81A	0.000	0.000	0.064	0.000	0.000
	T121A	0.305	0.493	0.125	0.002	0.286
	V157A	0.630	0.699	0.000	0.001	0.015
CrtM	F26A	0.009	0.006	0.001	0.242	0.040
	W38A	0.689	0.331	0.014	0.411	0.288
	F233S	0.019	0.101	0.000	0.074	0.001
Y81A	T121A	0.449	0.471	0.110	0.002	0.285
Y81A	V157A	0.833	0.188	0.003	0.001	0.015
T121A	V157A	0.165	0.043	0.012	0.003	0.777
F26A	W38A	0.885	0.116	0.004	0.136	0.014
F26A	F233S	0.000	0.044	0.009	0.366	0.391
W38A	F233S	0.253	0.781	0.210	0.814	0.694
Y81A	F26A	0.006	0.051	0.929	0.258	0.040
Y81A	W38A	0.675	0.461	0.244	0.414	0.288
Y81A	F233S	0.020	0.014	0.101	0.080	0.001
T121A	F26A	0.930	0.929	0.296	0.369	0.260
T121A	W38A	0.835	0.768	0.744	0.468	0.604
T121A	F233S	0.911	0.832	0.762	0.169	0.411
V157A	F26A	0.276	0.480	0.550	0.27	0.526
V157A	W38A	0.783	0.781	0.107	0.426	0.699
V157A	F233S	0.064	0.012	0.001	0.112	0.125

Supplementary Table 5

Mutations found in the CrtI variants.

Variants	Nucleotide and amino acid substitutions (nucleotide substitutions not followed by an amino acid substitution in brackets are silent)
$CrtI_{mut1}$	A195G, A594G, T1016C (**F339S**)
$CrtI_{mut2}$	A911G (**N304S**)
$CrtI_{mut4}$	T1015C (**F339L**), G1183A (**D395N**)
$CrtI_{mut6}$	A682G (**I228V**), A1012G (**I338V**)
$CrtI_{mut7}$	T144C, A1012G (**I338V**)
$CrtI_{mut8}$	A911G (**N304S**), T1017C

Supplementary Table 6

Carotenoid production levels in *E. coli* reported in the recent literature

Reference	Target	Carotenoid genes/ operons	Booster genes co-expressed	Production Titer (µg gDCW^{-1})
Wang 1999[27]	Astaxanthin	*crtE*[a], *crtWZYIB*[b]	-	33
Scaife 2012[28]	β-Carotene	*crtEYIB*[c]	-	85
Matthews 2000[29]	Lycopene	*crtEIB*[a]	-	160
Matthews 2000[29]	Zeaxanthin	*crtEYIBZ*[a]	-	186
Kajiwara 1997[25]	Lycopene	*crtEIB*[a]	-	228
Ruther 1997[30]	Zeaxanthin	*crtEYIBZ*[a]	-	289
Kajiwara 1997[25]	β-Carotene	*crtEYIB*[a]	-	488
Matthews 2000[29]	Zeaxanthin	*crtEYIBZ*[a]	*dxs*	526
Scaife 2012[28]	Canthaxanthin	*crtEYIB*[c], *crtW*[d]	-	570
Kang 2005[31]	Lycopene	*crtEIB*[c]	*ipiHp*	600
Harada 2009[26]	Astaxanthin	*crtW*[e], *crtEYIB*[a]	-	790
This study	C_{50}-Astaxanthin	$fds_{Y81A,V157A}$, $crtM_{F26A,W38A,F233S}$, $crtI^{a}_{N304P}$, *crtY*[a], *crtWZ*[e]	-	920
Kajiwara 1997[25]	Lycopene	*crtEIB*[a]	*ipiHp*	1,029
Kajiwara 1997[25]	β-Carotene	*crtEYIBZ*[a]	*ipiHp*	1,310
Scaife 2012[28]	Canthaxanthin	*crtEYIB*[c], *crtW*[d]	-	1,326
Matthews 2000[29]	Lycopene	*crtEIB*[a]	*dxs*	1,333
Wang 1999[27]	Astaxanthin	*crtE*[a], *crtWZYIB*[b]	*gps, idi*	1,419
Harada 2009[26]	Astaxanthin	*crtW*[e], *crtEYIB*[a]	*idi, aacl,* mevalonate pathway genes	1,470
Scaife 2009[32]	Astaxanthin	*crtEYIB*[c], *crtZ*[c], *crtW*[f]	-	1,990
Kang 2005[31]	Lycopene	*crtEIB*[c]	*appY, dxs*	4,700
Zelcbuch 2013[33]	Astaxanthin	*crtEBI*[c], *lcy-b*[g], *crtW*[h], *crtZ*[a]	*idi, dxs*	5,800
Yuan 2006[34]	β-Carotene	*crtEYIB*[i]	*dxs, idi, ispB, ispDF*	6,000
Wang 2009[35]	Lycopene	*crtEIB*[c]	*dxs, idi*	9,000
Scaife 2012[28]	Canthaxanthin	*crtEYIB*[c], *crtW*[d]	*idi, dxs, dxr*	11,700
Alper 2008[36]	Lycopene	*crtEIB*[c]	*dxs, idi, ispDF, del(gdh/aceE/yjiDp)*	18,000
Yoon 2006[34]	Lycopene	*crtEIB*[c]	*ipiHP,* mevalonate pathway genes	22,000
Scaife 2012[28]	Canthaxanthin	*crtEYIB*[c], *crtW*[d]	*idi, dxs, dxr,* optimized growth medium	78,200

[a]*Pantoea ananatis*, [b]*Agrobacterium aurantiacum*, [c]*Pantoea agglomerans*, [d]*Anabaena variabilis*, [e]*Brevundimonas* sp. SD212. [f]*Nostoc* sp. PCC 7120. [g]*Solanum lycopersicum*. [h]*Nostoc sphaeroides*, [i]*Pantoea stewartii*

Supplementary Table 7

Genes used in this study.

Gene	Gene product	Source organism	Ref.	Accession no.
**Isoprenyl diphosphate synthases**				
fds	Farnesyl diphosphate synthase	*G. stearothermophilus*	5	D13293
crtE	Geranylgeranyl diphosphate synthase	*P. ananatis*	39	D90087
idi	Isopentenyl diphosphate synthase	*E. coli*	27	AF119715
**Carotenoid backbone synthases**				
crtM	4,4'-Diapophytoene synthase	*S. aureus*	21	YP_501333<br>Mutation D5N
crtB	Phytoene synthase	*P. ananatis*	39	D90087
**Carotenoid desaturases**				
crtN	4,4'-Diapophytoene desaturase	*S. aureus*	21	YP_501332
crtI	Phytoene desaturase	*P. ananatis*	39	D90087
**Carotenoid modification enzymes**				
crtY	Lycopene β-cyclase	*P. ananatis*	39	D90087
crtW	β-Carotene ketolase	*Brevundimonas* sp. SD212[11], codon optimized for *E. coli*	This study	AB727658
crtZ	β-Carotene hydroxylase	*Brevundimonas* sp. SD212[10], codon optimized for *E. coli*	This study	AB727659
crtNb	4, 4'-Diaponeurosporene oxidase (4, 4'-Diapolycopene oxidase)	*S. aureus*	20	AY841893<br>Also named as *crtOx*[40] or *crtP*[a41]

[a] The name *crtP* is already used for phytoene desaturase in cyanobacteria, so it would be confusing to use it here for a 4,4'-diaponeurosporene oxidase.

Supplementary Table 8

Plasmids used in this study. See Supplementary Fig. 11 for plasmid maps.

Name	Vector	Genes
**Plasmids for carotenoid backbone synthesis**		
pUC-$\textit{fds}_{variants}$	pUC18m	P_{lac}-$\textit{fds}_{variants}$
pAC-$\textit{fds}_{variants}$	pACmod	P_{lac}-$\textit{fds}_{variants}$
pUC-$\textit{crtM}_{variants}$	pUC18m	P_{lac}-$\textit{crtM}_{variants}$
pAC-$\textit{crtM}_{variants}$	pACmod	P_{lac}-$\textit{crtM}_{variants}$
pAC-$\textit{fds}_{variants}$-$\textit{crtM}_{variants}$	pACmod	P_{lac}-$\textit{fds}_{variants}$, P_{lac}-$\textit{crtM}_{variants}$
pAC-$\textit{fds}_{Y81A,V157A}$-$\textit{crtM}_{F26A,W38A,F233S}$-*idi*	pACmod	P_{lac}-$\textit{fds}_{Y81A,V157A}$, P_{lac}-$\textit{crtM}_{F26A,W38A,F233S}$, P_{lac}-*idi*
pAC-$\textit{fds}_{variants}$-*idi*	pACmod	P_{lac}-$\textit{fds}_{variants}$, P_{lac}-*idi*
**Screening plasmids for directed evolution**		
pAC-*crtE-crtB-crtI-idi*	pACmod	P_{lac}-*crtE-crtB-crtI*, P_{lac}-*idi*
pAC-*crtE-crtI-idi*	pACmod	P_{lac}-*crtE-crtI,* P_{lac}-*idi*
pAC-*crtN-idi*	pACmod	P_{lac}-*crtN*, P_{lac}-*idi*
pAC-*crtN-crtNb-idi*	pACmod	P_{lac}-*crtN*, P_{lac}-*crtNb-idi*
**Plasmids for desaturase and modification enzymes**		
pUCara-$\textit{crtI}_{variants}$	pUCara	P_C-*araC*, P_{BAD}-$\textit{crtI}_{variants}$
pUCara-$\textit{crtI}_{variants}$-*crtY*	pUCara	P_C-*araC*, P_{BAD}-$\textit{crtI}_{variants}$-*crtY*
pUCara-$\textit{crtI}_{variants}$-*crtY-crtW*	pUCara	P_C-*araC*, P_{BAD}-$\textit{crtI}_{variants}$-*crtY-crtW*
pUCara-$\textit{crtI}_{variants}$-*crtY-crtZ*	pUCara	P_C-*araC*, P_{BAD}-$\textit{crtI}_{variants}$-*crtY-crtZ*
pUCara-$\textit{crtI}_{variants}$-*crtY-crtW-crtZ*	pUCara	P_C-*araC*, P_{BAD}-$\textit{crtI}_{variants}$-*crtY-crtW-crtZ*

$\textit{fds}_{variants}$: *fds*, $\textit{fds}_{Y81A}$, $\textit{fds}_{T121A}$, $\textit{fds}_{V157A}$, $\textit{fds}_{Y81A,T121A}$, $\textit{fds}_{Y81A,V157A}$, $\textit{fds}_{Y81A,\ T121A,\ V157A}$, $\textit{fds}_{Y81M}$

$\textit{crtM}_{variants}$: *crtM*, $\textit{crtM}_{F26A}$, $\textit{crtM}_{W38A}$, $\textit{crtM}_{F233S}$, $\textit{crtM}_{F26A,W38A}$, $\textit{crtM}_{F26A,F233S}$, $\textit{crtM}_{W38A,F233S}$, $\textit{crtM}_{F26A,W38A,F233S}$

$\textit{crtI}_{variants}$: *crtI*, $\textit{crtI}_{N304S}$, $\textit{crtI}_{N304P}$, $\textit{crtI}_{mut1}$, $\textit{crtI}_{mut2}$, $\textit{crtI}_{mut4}$, $\textit{crtI}_{mut6}$, $\textit{crtI}_{mut7}$, $\textit{crtI}_{mut8}$

Supplementary Table 9

Primers used for cloning.

Primer name	Sequence<br>• Restriction site in bold<br>• Over-hang sequence for type II restriction enzyme is indicated with boxes<br>• Annealing sequence in lowercase<br>• Homology sequence used for SLIC is italicized
FDS-Y81A-F	GCTA**GCTCTTC**AGCGtctttgatccatgatgatttgccgag
FDS-Y81A-R	CTAG**GCTCTTC**TCGCcgtatggatcatttcaatcgcgc
FDS-Y81M-F	GCTA**GCTCTTC**AATGtctttgatccatgatgatttgccgag
FDS-Y81M-R	CTAG**GCTCTTC**TCATcgtatggatcatttcaatcgcgc
FDS-I78G-F	TATA**GCTCTTC**AGGTcatacggcatctttgatccatgatg
FDS-I78G-R	TATA**GCTCTTC**AACCcatttcaatcgcgcaggcg
CrtM-F26A-F	TTTT**GCTCTTC**TGCGgacttgttaccagaagatcaaagaaaag
CrtM-F26A-R	TTTT**GCTCTTC**TCGCagcgtaagaaaagctttttgaatgtttcttc
CrtM-W38A-F	GCTA**GCTCTTC**AGCGgcaatttatgctgtgtgtcgtaaaattgatg
CrtM-W38A-R	CTAG**GCTCTTC**TCGCaaccgctttctttgatcttctggtaac
CrtM-F233S-F	GCTACT**GCTCTTC**GTCTagtattgaagcacaaccaatcatagaattag
CrtM-F233S-R	CTGATA**GCTCTTC**TAGAtactttgatttgatccataacatcttgaaaatc
CrtI-N304S	TTTT**GCTCTTC**TAGCtctctgtttgtgctctattttggtttgaatc
CrtI-N304S	TTTT**GCTCTTC**TGCTactcatgcgcttagtctgcag
CrtI-N304P	TTTT**GCTCTTC**TCCTtctctgtttgtgctctattttggtttgaatc
CrtI-N304P	TTTT**GCTCTTC**TAGGactcatgcgcttagtctgcag
pUCtopAC-SLIC-HindIII-F	*TTGACAGCTTATCATCGATAAGCTT*aggtttcccgactggaaagcg
pUCtopAC-SLIC-HindIII-R	*GTGATAAACTACCGCATTAAAGCTT*gtgaaataccgcacagatgcg
pUCtopAC-SLIC-BamHI-F	*GCGACCACACCCGTCCTGTGGATCC*aggtttcccgactggaaagcg
pUCtopAC-SLIC-BamHI-R	*ACGATGCGTCCGGCGTAGAGGATCC*gtgaaataccgcacagatgcg
pUCtopAC-SLIC-SalI-F	*GAGTCGCATAAGGGAGAGCGTCGAC*aggtttcccgactggaaagcg
pUCtopAC-SLIC-SalI-R	*AAGGCTCTCAAGGGCATCGGTCGAC*gtgaaataccgcacagatgcg
pBAD-vecF	TTTT**CCATGG**cgctttccagtcgggaaacct
pBAD-vecR	TTTT**gaattc**tctagactcgagaggag
pBAD-insF	TTTT**CCATGG**ttatgacaacttgacggctacatcattc
pBAD-insR	TTTT**GAATTC**aaacgggtatggagaaacagtagag
crtI-F	TTTT**CTCGAG**AGGAGGTTACAAAatgaaaccaactacggtaattggt
crtI-R	TTTT**GGGCCC**tcaaatcagatcctccagcatcaaac
crtY-F	CTGATA**GCTCTTC**TGTGTAGGAGGATTACAAAatgcaaccgcattatgatctgattctc
crtY-R	GCTACT**GCTCTTC**GGCA**ACTAGT**ttaacgatgagtcgtcataatggcttg
crtIdownstream-F	CTGATA**GCTCTTC**GTGCtgatgcggtattttctccttacgc
crtIdownstream-R	GCTACT**GCTCTTC**GCAC**gggccc**tcaaatcagatcc

Supplementary Table 10

Plasmid combinations used in each experiment

Figure	Plasmid 1 (pAC-based)	Plasmid 2 (pUC-based)
Fig. 2a	pAC-fds_{Y81A}-$crtM_{F26A,W38A}$	pUCara
Fig. 2b	pAC-fds_{Y81A}-$crtM_{F26A,W38A}$	pUCara-*crtI-crtY-crtW-crtZ*
Fig. 3a	pAC-*crtE-crtB-crtI-idi*	pUC-$fds_{variants}$
Fig. 3c	pAC-*crtE-crtI-idi*	pUC-$crtM_{variants}$
	pAC-*crtN-idi*	pUC-$crtM_{variants}$
Fig. 3d	pAC- fds_{Y81A}	pUC-$crtM_{variants}$
Fig. 4a	pAC-$fds_{variants}$	pUC-$crtM_{variants}$
Fig. 5	pAC-$fds_{variants}$	pUC-$crtM_{variants}$
Fig. 6	pAC-$fds_{Y81A,V157A}$-$crtM_{F26A,W38A,F233S}$	pUCara-$crtI_{variants}$
Fig. 7a	pAC-$fds_{Y81A,T121A,V157A}$-$crtM_{F26A,F233S}$	pUCara-$crtI_{N304P}$-*crtY*
	pAC-fds_{Y81M}-$crtM_{F26A,W38A}$	pUCara-*crtI-crtY*
Fig. 7b	pAC-$fds_{Y81A,T121A,V157A}$-$crtM_{F26A,F233S}$	pUCara-$crtI_{N304P}$-*crtY-crtZ*
	pAC-$fds_{Y81A,T121A,V157A}$-$crtM_{F26A,F233S}$	pUCara-$crtI_{N304P}$-*crtY-crtW*
	pAC-$fds_{Y81A,T121A,V157A}$-$crtM_{F26A,F233S}$	pUCara-$crtI_{N304P}$-*crtY-crtW-crtZ*
	pAC-fds_{Y81M}-$crtM_{F26A,W38A}$	pUCara-*crtI-crtY-crtZ*
	pAC-fds_{Y81M}-$crtM_{F26A,W38A}$	pUCara-*crtI-crtY-crtW*
	pAC-fds_{Y81M}-$crtM_{F26A,W38A}$	pUCara-*crtI-crtY-crtW-crtZ*
Supplementary Fig. 3a	pAC-*crtE-crtB-crtI-idi*	pUC18m
	pAC-*crtE-crtB-crtI-idi*	pUC-$fds_{variants}$
	pAC-*crtM-crtN*	pUC18m
	pAC-*crtM-crtN*	pUC-$fds_{variants}$
Supplementary Fig. 5b	pAC-*crtN-crtNb-idi*	pUC-$crtM_{variants}$
	pAC-*crtE-crtI-idi*	pUC-$crtM_{variants}$
Supplementary Fig. 5d	pAC-*crtN-idi*	pUC-$crtM_{variants}$
Supplementary Fig. 5e	pAC-*crtE-crtI-idi*	pUC-$crtM_{variants}$
Supplementary Fig. 5f	---	pUC-$crtM_{variants}$
Supplementary Fig. 5g	pAC-*crtE*	pUC-$crtM_{variants}$
Supplementary Fig. 6	pAC-$fds_{I78G,Y81A}$-*idi*	pUC-$crtM_{F26A,W38A}$
	pAC-$fds_{I78G,Y81A}$-*idi*	pUC-$crtM_{F26A,W38A,F233S}$
Supplementary Fig. 8a	pAC-fds_{Y81A}-$crtM_{F26A,W38A}$	pUC-*crtI library*
	pAC-$fds_{Y81A,V157A}$-$crtM_{F26A,W38A,F233S}$	pUC-*crtI library*
Supplementary Fig. 8b,d	pAC-$fds_{Y81A,V157A}$-$crtM_{F26A,W38A,F233S}$	pUCara-$crtI_{variants}$
Supplementary Fig. 8c	pAC-fds_{Y81M}-$crtM_{F26A,W38A}$	pUCara-$crtI_{variants}$
Supplementary Fig. 9	pAC-*crtE-crtB*	pUCara-*crtI-crtY-crtW-crtZ*
	pAC-$fds_{Y81A,V157A}$-$crtM_{F26A,W38A,F233S}$	pUCara-$crtI_{N304P}$-*crtY-crtW-crtZ*
Supplementary Fig. 10a	pAC-fds_{Y81M}-$crtM_{F26A,W38A}$	pUCara-*crtI-crtY*
Supplementary Fig. 10b	pAC-fds_{Y81M}-$crtM_{F26A,W38A}$	pUCara-*crtI -crtY-crtZ*
Supplementary Fig. 10c	pAC-fds_{Y81M}-$crtM_{F26A,W38A}$	pUCara-*crtI -crtY-crtW*
Supplementary Fig. 10d	pAC-fds_{Y81M}-$crtM_{F26A,W38A}$	pUCara-*crtI -crtY-crtW-crtZ*
Supplementary Fig. 10e	pAC-$fds_{Y81A,V157A}$-$crtM_{F26A,W38A,F233S}$	pUCara-$crtI_{N304P}$ *-crtY*
Supplementary Fig. 10f	pAC-$fds_{Y81A,V157A}$-$crtM_{F26A,W38A,F233S}$	pUCara-$crtI_{N304P}$ *-crtY-crtZ*
Supplementary Fig. 10g	pAC-$fds_{Y81A,V157A}$-$crtM_{F26A,W38A,F233S}$	pUCara-$crtI_{N304P}$ *-crtY-crtW*
Supplementary Fig. 10h	pAC-$fds_{Y81A,V157A}$-$crtM_{F26A,W38A,F233S}$	pUCara-$crtI_{N304P}$ *-crtY-crtW-crtZ*

$fds_{variants}$: *fds*, fds_{Y81A}, fds_{T121A}, fds_{V157A}, $fds_{Y81A,T121A}$, $fds_{Y81A,V157A}$, $fds_{Y81A, T121A, V157A}$, fds_{Y81M}

$crtM_{variants}$: *crtM*, $crtM_{F26A}$, $crtM_{W38A}$, $crtM_{F233S}$, $crtM_{F26A,W38A}$, $crtM_{F26A,F233S}$, $crtM_{W38A,F233S}$, $crtM_{F26A,W38A,F233S}$

$crtI_{variants}$: *crtI*, $crtI_{N304S}$, $crtI_{N304P}$, $crtI_{mut1}$, $crtI_{mut2}$, $crtI_{mut4}$, $crtI_{mut6}$, $crtI_{mut7}$, $crtI_{mut8}$

Supplementary Table 11

Molar extinction coefficients (ε) of the C_{50}-carotenoids.

Name	Description[a]	ε (M^{-1} cm^{-1})	Wavelength (nm)	Source
C_{50}-Phytoene (**7**)	C_{50} skeleton (n=3)	50,000	286	Ref. 8
C_{50}-Carotene (n=7)	2-step desaturated (n=7)	138,000	400	Ref. 8
C_{50}-Phytofluene	3-step desaturated (n=9)	147,000	438	Ref. 8
C_{50}-Neurosporene	4-step desaturated (n=11)	185,000	472	Ref. 8
C_{50}-Lycopene (**8**)	6-step desaturated (n=15)	190,000	527	Ref. 13
C_{50}-β-Carotene (**9**)	n = 13+2(beta)	183,105	501	Ref. 37
C_{50}-Zeaxanthin (**10**)	n = 13+2(beta)	180,000	518	Ref. 13
C_{50}-Canthaxanthin (**11**)	n = 13+2(beta)+2(keto)	190,000	527	ε taken from C_{50}-astaxanthin
C_{50}-Astaxanthin (**12**)	n = 13+2(beta)+2(keto)	190,000	527	Ref. 13

[a]: n represents the number of conjugated double bonds

Supplementary Note 1
Design of the biosynthetic pathway for C_{50}-astaxanthin

1.1. Basic Design

The pathway for C_{50}-astaxanthin (**12**) was designed by mirroring the natural astaxanthin (**6**) pathway (**Fig. 1**). Instead of condensing two molecules of C_{20}PP (geranylgeranyl diphosphate) to make C_{40} backbone (phytoene, **1**), a C_{50} backbone (C_{50}-phytoene, **7**) is synthesized by the condensation of two molecules of C_{25}PP (geranylfarnesyl diphosphate). Next, the C_{50} backbone (**7**) undergoes six desaturation steps, thereby developing a chromophore with 15 conjugated double bonds. The resultant C_{50}-lycopene (**8**) is then cyclized, hydroxylated, and ketolated by the enzymes recruited from natural astaxanthin (**6**) pathways. Altogether, the pathway consists of six enzymes catalyzing a total of fifteen chemical transformations.

1.2. Description of the enzymatic steps

Escherichia coli synthesizes C_{15}PP (farnesyl diphosphate) by the consecutive condensation of two molecules of isopentenyl diphosphate (IPP) with dimethylallyl diphosphate (DMAPP), both of which are provided by the 2-*C*-methyl-D-erythritol 4-phosphate (MEP) pathway. From this C_{15}PP, our pathway produces C_{50}-astaxanthin via the following steps:

i) *A two-step prenyl transfer reaction to create $C_{25}PP$:* Naturally-occurring C_{25}PP synthases exist in some bacteria and archaea[1-3]. However, they synthesize C_{20}PP in approximately equal proportion with C_{25}PP. On top of this, the expression of the *Aeropyrum pernix* C_{25}PP synthase gene accumulates only C_{20}PP in *Escherichia coli*[4], which is probably due to a suboptimal working temperature. Instead, we chose the specificity-shifting mutant of farnesyl diphosphate synthase (FDS_{Y81A}) from *Geobacillus stearothermophillus*[5]. Expression of FDS_{Y81A} in *E. coli* resulted in the accumulation of C_{25}PP, but only as a mixture with C_{15}PP and C_{20}PP[6]. Further engineering was required to convert this variant into a more specific and efficient C_{25}PP synthase (**Fig. 3a**).

ii) *A one-step head-to-head condensation of the precursors to make C_{50}-phytoene (7):* There are two types of bacterial carotenoid backbone synthases in nature: CrtM for C_{30} backbone and CrtB for C_{40} backbone (**1**). Previously, we discovered that the F26A/W38A mutant of *Staphylococcus aureus* CrtM produced small but detectable amounts of C_{50} backbone[6] (**7**). In this paper, we further evolved this variant for improved preference for C_{50} backbone synthesis (**Fig. 3c**).

iii) *A six-step desaturation for chromophore formation to make C_{50}-lycopene (**8**):* Natural two-, three-, four-, and five-step carotenoid desaturases are known, but no six-step desaturases have been discovered in nature. Because the C_{40} backbone desaturase (CrtI) from *Pantoea ananatis* showed a detectable level of C_{50} backbone desaturation[7], we decided to evolve it into an efficient six-step desaturase (**Fig. 6a**).

iv) *A two-step cyclization to make C_{50}-β-carotene (9):* Cyclization is widespread in C_{40} carotenoid pathways but not observed in the C_{30} pathway at all. However, lycopene cyclases are known to be 'locally-specific' enzymes that recognize only a particular part (locus) of their substrates[8]. Therefore, they can accept a variety of non-cognate substrates, including C_{35} carotenoids. Because C_{50} carotenoids possess the same loci for cyclization as C_{40} carotenoids[9] (ψ- and 7,8-dihydro-ψ ends), we reasoned that the natural C_{40} carotenoid cyclase (CrtY from *P. ananatis*) had an excellent probability of acting on C_{50}-lycopene (**8**) to synthesize C_{50}-β-carotene (**9**). We found this was indeed the case (**Fig. 7a**).

v) *A two-step hydroxylation to produce C_{50}-zeaxanthin (10):* In natural C_{40} carotenoid pathways, zeaxanthin (**4**) is formed by enzymatic hydroxylation of two specific positions (3- and 3'-moieties) of β-carotene (**3**). As was often the case with other modification enzymes, we later found these steps in the C_{50} pathway could be fulfilled simply by recruiting the β-carotene 3-hydroxylase (CrtZ) from an astaxanthin-producing microbe (**Fig. 7b**). The most promising CrtZ seemed to be the one from *Brevundimonas* sp. SD212: literature indicated that this had the least substrate specificity among the known homologues[10]. Due to its high GC-content, we decided to codon-optimize the *Brevundimonas* CrtZ for expression in *E. coli*.

vi) *A two-step ketolation to produce C_{50}-canthaxanthin (11):* In natural (C_{40}) carotenoid pathways, canthaxanthin is formed by enzymatic ketolation at the 4 and 4'positions of β-carotene (**3**) (**Fig. 7b**). β-Carotene 4-ketolase (CrtW) from *Brevundimonas* sp. SD212 seemed to have the lowest degree of substrate specificity among the known CrtW homologues[11]. Due to its high GC-content, we decided to codon-optimize the *Brevundimonas* CrtW for *E. coli*.

vii) *The routes for C_{50}-astaxanthin (12):* The last four steps catalyzed by CrtZ and CrtW are known to constitute a representative example of a so-called 'matrix pathway', where eight different intermediates can be created (See **Fig. 1** and **Supplementary Fig. 1**). It is not known whether the path from C_{50}-β-carotene (**9**) to C_{50}-astaxanthin (**12**) [or the path from β-carotene (**3**) to astaxanthin(**6**)] proceeds in a defined or random (matrix) sequence.

1.3. Nomenclature and issues on trivial names of C_{50} carotenoids

As shown in **Fig. 1**, the C_{50}-astaxanthin (**12**) pathway parallels the astaxanthin (**6**) pathway. Considering the biochemical steps to acyclic, cyclic and oxo-cyclic C_{50} carotenoids toward C_{50}-astaxanthin (**12**) and the hundreds of other C_{50} carotenoids we would in principle be able to biosynthesize in the future, we propose that the most convenient nomenclature rule for C_{50} carotenoids is that named simply by adding prefix "C_{50}-" to the trivial names of their respective, natural C_{40} counterparts. We have adopted this rule for the six C_{50} carotenoids (**7-12**) reported in this paper, as shown in **Fig. 1** and **Supplementary Fig. 1**.

On the skeletal structure of C_{50} carotenoids. Historically, there have been various ways to name C_{50} carotenoids, and we anticipate some confusion in the trivial naming of carotenoids with unique skeletons. Years ago, synthetic chemists synthesized the "C_{50}-versions" of β-carotene (**9**), zeaxanthin (**10**), and astaxanthin (**12**). Based on their sub-structural elements, these carotenoids were named decapreno-β-carotene[12], decaprenozeaxanthin[13], and decaprenoastaxanthin[13], respectively. On the other hand, some bacteria are known to biosynthesize a different type of C_{50}-carotenoids[14] formed by the attachment of an isopentenyl (C_5) unit to each end of lycopene (C_{40}), yielding cyclic and acyclic C_{50} carotenoids. Although they are structurally different from the carotenoids the synthetic chemists and we have created, some of them are also called 'decapreno'-carotenoids. In our previous work, we referred to the C_{50} backbone (**7**) as 1,1'-diisopentenylphytoene[6]. This nomenclature rule is applicable to acyclic C_{50} carotenoids with zero- to six- desaturation step numbers, but not to those with seven- and eight-step numbers or to cyclized ones. This drove us to refer to C_{50} carotenoids based on their structural similarity and parallel biosynthesis to their C_{40} analogues.

Confusion in desaturase step numbers. In the rule above, C_{50}-lycopene (**8**) is the six-step desaturation product of C_{50} backbone (C_{50}-phytoene, **7**). Although it shares the same terminal structures (ψ-ends) with its natural C_{40} counterpart, lycopene (**2**), its chromophore is longer than that of lycopene: C_{50}-lycopene has 15 conjugated double bonds in its chromophore, while (C_{40}) lycopene is a four-step desaturation product of phytoene (**1**), and it has 11 conjugated double bonds in its chromophore. This simple rule is applicable to carotenoids with other unnatural backbone sizes. For instance, the hypothetical 8-step desaturation product of C_{60} backbone would be called C_{60}-lycopene, and it would have 19 conjugated double bonds in its chromophore.

One problem with this nomenclature rule, however, is the inconvenience of naming the 1-, 2-, 3-, 4-, and 5-step desaturation products of (acyclic) C_{50} backbone (**7**). In the C_{40} pathway, the 1-, 2-, 3-, 4-, 5-, and 6-step desaturation products of phytoene (**1**) are called phytofluene, ζ-carotene, neurosporene, lycopene, 3,4-didehydrolycopene, and 3,4,3',4'-tetradehydrolycopene, respectively. This system cannot directly apply to C_{50} carotenoids because there exist two possible additional desaturation products of C_{50} backbone (**7**). To deal with these problems, we propose to name the C_{50} backbone (**7**) (condensation product of two molecules of C_{25}PPs) as C_{50}-phytoene. The other acyclic C_{50}-carotenes are named as follows: "C_{50}-carotene (n=5)," "C_{50}-carotene (n=7)," "C_{50}-carotene (n=9)," "C_{50}-carotene (n=11)," "C_{50}-carotene (n=13)," "C_{50}-carotene (n=17)," and "C_{50}-carotene (n=19)," for the 1-, 2-, 3-, 4-, 5-, 7-, and 8-step desaturation products of C_{50}-phytoene (n refers to the number of conjugated double bonds in the chromophore) (**Supplementary Fig. 1**).

In addition, we propose the parallel use of aforementioned "terminal structure nomenclature rule" to the carotenoids except for phytoene (C_{50}-phytofluene, C_{50}-ζ-carotene, C_{50}-neurosporene, C_{50}-lycopene, C_{50}-didehydrolycopene, and C_{50}-tetradehydrolycopene (see **Supplementary Fig. 1**). Here, C_{50}-carotene (n=5) and C_{50}-carotene (n=7) do not have their names with this rule [same is true for imaginary C_{60}-carotenes (n=5, 7, 9, 11)].

Supplementary Note 2
The number of biochemically possible carotenoids that can be produced by the combinatorial expression of six promiscuous enzymes along the C_{50}-astaxanthin pathway

According to the literature, each of the six enzymes along the path from $C_{15}PP$ to C_{50}-astaxanthin (**12**) possesses considerable tolerance to alternative substrates:

1. FDS_{Y81A} synthesizes $C_{15}PP$, $C_{20}PP$ and $C_{25}PP$[5]. The I78G mutation further increases the number of consecutive condensation steps, allowing the enzyme to synthesize $C_{30}PP$[15].
2. $CrtM_{F26A,W38A}$ synthesizes C_{30}, $C_{35,}$ C_{40}, C_{45}, and C_{50} backbones by the conjugation of two molecules of $C_{15}PP$, $C_{20}PP$ and $C_{25}PP$[6].
3. CrtI desaturates single bonds in a step-wise fashion, elongating the system of conjugated double bonds by two for each step[7,16]. It is known that in each step, CrtI only acts on the saturated sites adjacent to the developing chromophore[16]. In other words, CrtI desaturates only positions that would increase the size of the conjugated system comprising the chromophore.
4. CrtY cyclizes not only the ψ-end group but also the 7,8-dihydro-ψ end group[9]. In addition, we know that *P. ananatis* CrtY cyclizes carotenoids with different backbones [such as C_{35}[17], C_{30}[18], and C_{50} carotenoids (this work)]. That is, CrtY acts on C_{15}-, C_{20}-, and C_{25}-"halves" of carotenoids.
5. CrtW oxidizes position 4 of β-end groups with or without 3-keto groups[11]. We confirmed herein that CrtW acts on both sides of C_{50} carotenoids, indicating β-cyclized halves are good substrates for CrtW, irrespective of their size, from C_{15} to C_{25}. The same applies to CrtZ, the hydroxylase that acts on position 3 of β-end groups[10].

From the information above, we calculated the number of possible carotenoids that could be created by combinatorial expression of the six enzymes above, for each of carotenoid backbone (**Supplementary Table 1** and **Supplementary Fig. 2**; see below for the explanation of the calculation). A total of 642 possible carotenoids can be biosynthesized starting from a pool of the three precursors, $C_{15}PP$, $C_{20}PP$, and $C_{25}PP$. If $C_{30}PP$ is additionally considered, the number of possible carotenoids reaches 929. Note that even the natural (C_{40}) astaxanthin pathway can harbor 78 different compounds. Heterologous expression of the natural biosynthetic genes required for formation of astaxanthin (**6**) indeed results in the frequent accumulation of multiple compounds other than astaxanthin[10,11,19].

Below we provide the explanation of calculation in **Supplementary Table 1**. The alphabets (A-E) in **Supplementary Table 1** corresponds to that in this section.

A. The number of acyclic carotenoids for a given backbone size:

For symmetrical backbones (C_{30}, C_{40} and C_{50}), diversity at the level of desaturation can be calculated using the formula for combinations without permutation ("x-Choose-y"):

$$\underline{{}_{n+r-1}C_r = (n+r-1)! / r!\,(n-1)!}$$

where

n: maximum number of desaturation steps possible for the half backbone (see **Supplementary Fig. 2a**).

r: number of objects in combination. We use 2, for both sides of the backbone

For example, for the C_{30} backbone, there are three kinds of steps: 0, 1 or 2 steps (see **Supplementary Fig. 2a**). So the total number of steps (*n*) will be 3. There are two "independent" sides of the backbone (two half backbones) to consider, so *r* should be 2.

The total number of carotenoids that can arise from desaturation of asymmetric carotenoid backbones (such as C_{35} and C_{45}) can be calculated by multiplying together the step numbers (counting zero) for each half.

B. The number of monocyclic carotenoids (for a given backbone size):

There are two types of end groups that CrtY can act upon: the ψ and 7,8-dihydro-ψ ends (see **Supplementary Fig. 2b**). Note that the conjugated system must extend to either end for cyclization to occur.

For carotenoids with symmetric backbones: the number of monocyclic carotenoids can be calculated as follows:

<u>[the number of desaturation steps for the half backbone] × 2</u>

Explanation: the factor of 2 accounts for the "choice" of ψ or dihydro-ψ end groups as substrates for cyclization. The number of steps for the half backbone refers to the desaturation level of the non-cyclized side.

For asymmetric backbones:

<u>[the sum of the maximum number of desaturation steps for each half] x 2</u>

C. The number of bicyclic carotenoids (for given backbone size):

For symmetric backbones, there are <u>3</u> kinds of bicyclic carotenoids, while there are <u>4</u> for asymmetric bicyclic carotenoids (see **Supplementary Fig. 2c**)

D. The number of monocyclic xanthophylls (for given backbone size):

Starting from monocyclic precursors, there exist three types of oxidized products by the action of ketolase and hydroxylase: 3-hydroxylated, 4-ketolated, and 3-hydroxylated/4-ketolated. Therefore, the total number of possible monocyclic xanthophylls for a given backbone size can be obtained by multiplying the number of monocyclic carotenoids by <u>3</u> (see **Supplementary Fig. 2d**).

E. The number of bicyclic xanthophylls (for given backbone size):

As was discussed in **Supplementary Note 1** and **Supplementary Fig. 1**, there are 9 different oxidation patterns for each symmetric bicyclic carotenoid (see **Supplementary Fig. 2e**). On the other hand, there exist 15 different oxidation patterns for each asymmetric bicyclic carotenoid (see **Supplementary Fig. 2f**).

For carotenoids with symmetric backbones (C_{30}, C_{40} and C_{50}), there exist 3 bicyclic products, 2 of which are symmetric and the other asymmetric (see **Supplementary Fig. 2c**). Therefore, the number of possible oxidized products for each symmetric bicyclic substrate is:

$$2 \times 9 + 1 \times 15 = 33$$

For the asymmetric backbones (C_{35}, C_{45}), there exist 4 bicyclic products, all of which are asymmetric (see **Supplementary Fig. 2c**). Therefore, the number of possible oxidized products for each symmetric bicyclic substrate is:

$$4 \times 15 = 60$$

Supplementary Note 3
Directed evolution of farnesyl diphosphate synthase (FDS) for improved C_{25}PP precursor supply

Directed evolution of FDS_{Y81A}. A library of random point mutants of FDS_{Y81A} was created by error-prone PCR and cloned into a pUC-based vector. The resultant plasmid library was transformed into *E. coli* cells harboring pAC-*crtE-crtB-crtI-idi* (see **Fig. 3a**), which were then plated on LB-agar to form colonies. The rationale was that FDS variants with improved activity for converting C_{20}PP into C_{25}PP would more fully deplete the lycopene precursor C_{20}PP, resulting in paler colonies (**Fig. 3a**). We visually screened approximately 600 colonies, and found three colonies (named FDS_{m1}, FDS_{m2} and FDS_{m3}) that appeared much paler than those expressing the parent FDS_{Y81A}.

C_{20}PP consumption assay. We scored the *in vivo* C_{40} carotenoid accumulation to compare the variants' ability of C_{20}PP consumption (**Supplementary Fig. 3a**) of the three FDS variants. *E. coli* harboring plasmid pAC-*crtE-crtB-crtI-idi* accumulates lycopene to about 300 μg $gDCW^{-1}$. Additional expression of FDS_{Y81A} on a pUC vector resulted in decreased lycopene production (~100 μg $gDCW^{-1}$) by diversion of the intermediate C_{20}PP from the lycopene pathway. As anticipated, additional expression of each of the three isolated FDS variants resulted in even lower (0-30 μg $gDCW^{-1}$) levels of lycopene accumulation. This indicates that the three selected variants possess improved *in vivo* C_{20}PP consumption activity.

C_{15}PP consumption assay. We also tested the effect of FDS expression on the level of C_{30} carotenoid accumulation to compare the variants' abilities to consume C_{15}PP (**Supplementary Fig. 3a**). Here, transformation of pAC-*crtM-crtN* causes *E. coli* to accumulate ~200 μg $gDCW^{-1}$ of 4,4'-diaponeurosporene by way of C_{15}PP as precursor. Co-expression of FDS_{Y81A} completely abolished pigment accumulation, but co-expression of the 3 isolated mutants did not (**Supplementary Fig. 3a**). Thus, the 3 new FDS mutants appear to possess slightly compromised *in vivo* C_{15}PP consumption activity compared with FDS_{Y81A}.

Sequence analysis. The sequences of these clones are summarized in **Supplementary Table 2**. FDS_{m1} and FDS_{m3} contain only one amino acid substitution each: T121S and V157A, respectively. FDS_{m2} contains three additional amino acid substitutions, in addition to a different substitution at T121 (T121A) than found in FDS_{m1}. Because the C_{20}PP consumption activity of FDS_{m1} was not higher than FDS_{m2} (**Supplementary Fig. 3a**), we conclude that the three other mutations (H215R, P239T, F266L) probably do not contribute to improvement of C_{20}PP consumption activity.

Mutation mapped on crystal structure. When mapped onto the crystal structure of *S. aureus* FDS (**Supplementary Fig. 3b**), residues T121 and V157 were located on the "wall" of the substrate pocket.

We speculated that these mutations (T121A/S and V157A) enlarge the reaction pocket because they substitute smaller amino acids, thereby better accommodating larger substrates and products. Therefore, we chose to move forward with Ala instead of Ser for the substitution at residue 121. Altogether, we verified that Y81A, T121A and V157A were size-shifting substitutions in FDS.

In vitro product analysis of FDS variants. Each FDS variant was purified using a his-tag column and product distributions were evaluated *in vitro* (see **Methods**). [1-^{14}C]IPP and DMAPP were provided to the purified FDS variants, and the hydrolyzed products were analyzed using TLC autoradiography. Throughout this experiment, reaction times were limited so that <25% of the DMAPP was consumed, in order to obtain data representative of specificity under in vivo conditions of constant substrate supply. The molar ratio of IPP:DMAPP was set at 10:1, 5:1 or 1:1.

Results are shown in **Fig. 3b** and **Supplementary Fig. 4**. The molar ratio of IPP:DMAPP affected the product distribution: larger fractions of IPP resulted in larger fractions of longer products. Wild-type FDS produced only $C_{15}PP$ (100%) under all three IPP:DMAPP ratio, showing its stringent product specificity. On the other hand, most FDS variants showed relaxed specificity towards a larger product, and none of them yielded a single product. Four FDS variants with the Y81A mutation produced $C_{30}PP$ as the terminal product. Especially, $FDS_{Y81A,T121A}$ and $FDS_{Y81A,T121A,V157A}$ produced a relatively larger amount of $C_{30}PP$, under the condition of IPP:DMAPP = 10:1 (**Supplementary Fig. 4c**). $FDS_{Y81A,V157A}$ appeared to be the most selective $C_{25}PP$ producer in this *in vitro* experiment. However, the conditions of this experiment may differ in important ways from those in *E. coli* cytoplasm, and the *in vitro* product specificities of these purified FDS variants do not necessarily mirror the proportions of available carotenoid precursors in *E. coli*; see below. It should be noted:

(i) Although over-expressed, FDS variants are not the only providers of carotenoid precursors: an endogenous $C_{15}PP$ synthase, IspA, is also present. The product spectrum of FDS variants could be further shifted toward larger products, when $C_{15}PP$ was fed as a substrate. Also the $C_{15}PP$ produced by IspA could be used as a substrate for carotenoid synthase (CrtM) variants, but not only FDS variants.

(ii) Endogenous isoprenyl diphosphate consumers are available in the cell: IspB uses FPP as a substrate to synthesize octaprenyl diphosphate which are the precursor for quinones, and IspU also uses FPP to synthesize undecaprenyl diphosphates, the precursor for undecaprenyl phosphate, a lipid career for cell wall carbohydrate.

(iii) Carotenoid production experiments are performed in long-term cultures that reach late-stationary phase (typically 48-72 hours), to allow for carotenoid accumulation. The IPP/DMAPP ratio could change substantially over this course .

(iv) Finally, the FDS and CrtM variants form a ‘matrix pathway’: unlike the *in vitro* condition where the FDS variants are isolated, their conversion of $C_{15}PP$ to $C_{20}PP$ or $C_{20}PP$ to $C_{25}PP$ occurs in competition with a carotenoid synthase, which can potentially convert these precursors into

C_{30}–C_{50} carotenoid backbones. This type of matrix/competition experiment was not possible for us to replicate *in vitro*.

For instance, *in vitro*, $FDS_{Y81A,V157A}$ (FDS_{m3}) did not produce $C_{15}PP$ (**Supplementary Fig. 4**). However, this same variant apparently produced slightly more $C_{15}PP$ compared to FDS_{Y81A} in *E. coli* (**Supplementary Fig. 3a** bottom panel). Also, when $FDS_{Y81A,V157A}$ was co-expressed with various carotenoid synthase variants, asymmetric C_{40} carotenoids (condensation product of $C_{15}PP$ and $C_{25}PP$) accumulated (**Fig. 4a**). This means that the *E. coli* cells expressing FDS_{Y81A} clearly feeds $C_{15}PP$ to the carotenoid pathway, in addition to $C_{25}PP$.

Supplementary Note 4. Directed evolution of diapophytoene synthase (CrtM) for improved C_{50} synthase activity

Strategy for screening C_{50}-synthase activity. Previously, we had discovered some mutants of CrtM [the C_{30} backbone (diapophytoene) synthase from *S. aureus*] with detectable C_{50} backbone (**7**) synthase activity[6]. However, these mutants also synthesize C_{35}, C_{40} (symmetric C_{20}PP+C_{20}PP, and asymmetric C_{25}PP+C_{15}PP), and C_{45} backbones. To create a more specific C_{50} backbone synthase, we decided to conduct additional rounds of directed evolution on our CrtM variants. Because there is no simple medium- or high-throughput assay for C_{50} backbone synthesis, we decided instead to use our established colony color-based screens for C_{30}- and C_{40}-activites. We hypothesized that mutations that further shift the size specificity of a C_{50}-capable CrtM variant toward larger substrates might be obtained by searching for additional mutations that diminish C_{30} backbone synthase function but maintain C_{40} synthase function (**Supplementary Fig. 5a**). We hoped that these additional mutations could then be combined with previously-discovered ones to further shift the specificity of CrtM toward larger substrates and products.

$CrtM_{W38A}$ as a parent for directed evolution. As the parent for mutagenesis and directed evolution, we chose $CrtM_{W38A}$, a variant with both C_{30} and C_{40} backbone synthase activities[6] instead of $CrtM_{F26A,W38A}$, the variant with the best C_{50} synthase activity at the time. The latter enzyme exhibited very low C_{30} synthase activity, so it would have been difficult to screen for mutations that further decreased its C_{30} activity. In contrast, $CrtM_{W38A}$ is a 'generalist' mutant that retains wild-type C_{30} synthase activity.

Directed evolution of $CrtM_{W38A}$. Using error-prone PCR, we created a library of genes encoding variants of $CrtM_{W38A}$, which we cloned into a pUC-based vector. The plasmid library was co-transformed with pAC-*crtE-crtI-idi* (see left panel in **Fig. 3c**) into *E. coli* cells, which were plated on LB-agar. About 70% of the ~200 variant colonies screened had red pigmentation, indicating the desired retention of substantial C_{40} synthase activity. From among them, approximately 50 colonies were picked, pooled, and subjected to plasmid purification. This mixture of plasmids was then used as the template for the next round of PCR mutagenesis, which was followed by further screening for maintenance of C_{40} activity. Five successive rounds of this process were conducted to accumulate mutations apparently neutral to C_{40} function. Next, the resultant plasmid mixture was co-transformed with pAC-*crtN-idi* (see right panel in **Fig. 3c**) in a search for white colonies (indicating diminished C_{30} synthase activity). Three mutants conferring the desired white phenotype were isolated and named $CrtM_{g5L-1}$, $CrtM_{g5H-1}$, $CrtM_{g5H-2}$.

C_{40}- and C_{30}-activity of isolated CrtM variants. To test the activity of these mutants as C_{40} and

C_{30} synthases, the CrtM mutants were re-transformed into XL1-Blue cells harboring pAC-*crtE-crtI-idi* and pAC-*crtN-crtNb-idi* (here, CrtNb, a C_{30} carotenoid oxidase[20] that converts 4,4'-diaponeurosporene to 4,4'-diaponeurosporenal (an orange pigment) was additionally expressed to facilitate the visual screening). We reconfirmed that all three CrtM variants demonstrated the desired combination of reduced C_{30} synthase activity and undiminished C_{40} synthase activity (**Supplementary Fig. 5b**).

Sequence analysis. Sequencing of the three isolated CrtM variants (**Supplementary Table 3**) revealed that they all possess a mutation at F233 (F233S or F233L), and two of the three also have a previously reported size-shifting mutation (F26L)[21]. Previously, Umeno *et al.* performed site-saturation mutagenesis at position 26 of CrtM, and concluded that F26A was the best substitution for C_{40} synthase activity[6]. As a result, in addition to the W38A mutation in the parent, we decided to move forward with combinatorial testing of the F26A and F233S mutations.

Structural mapping of F233S mutation. Although F233S alone does not appear to change the specificity of CrtM, this mutation significantly shifts the product specificity of CrtM to C_{50} synthesis when combined with F26A and/or W38A (**Fig. 4** and **Supplementary Fig. 5d-g**). Located at the end of the active site cleft of CrtM, F233S is expected to further shift the size specificity of CrtM by enlarging the pocket (**Supplementary Fig. 5c**). This shifted specificity of F233S-containing variants is further emphasized when $C_{30}PP$ is supplied to it as a potential precursor for carotenoid synthesis. Upon co-expression with $FDS_{I78G,Y81A}$[15], $CrtM_{F26A,W38A,F233S}$ produced C_{55}-phytoene, the largest carotenoid backbone ever reported to have been biosynthesized (**Fig. 4a** and **Supplementary Fig. 6**).

Supplementary Note 5.
Regression analysis of carotenoid backbone titers vs mutations from the combinatorial expression of FDS and CrtM mutants

Because the true product distribution of FDS variants is extremely challenging to measure in living cells, we performed multiple linear regression on the carotenoid backbone titer data behind the bars in **Fig. 4a** (an 8×8 full-factorial experiment) in an effort to ascertain the contributions of the FDS and CrtM mutations and their interactions to the measured titers. Using the General Linear Model function in Minitab® v.16.2, we performed 5 separate regressions, one for each carotenoid backbone from C_{30} to C_{50} (see **Supplementary Table 4**), of the backbone titer vs. the set of 6 total amino acid substitutions in the FDS and CrtM variants (3 each) plus the 15 two-body (epistatic) interaction terms of the 6 substitutions. We were most interested in the resulting sets of *p*-values (from two-tailed F-tests) for each term in each model. For each carotenoid backbone model, we note there is at least one FDS-CrtM substitution interaction term (indicated by the bottom 9 rows containing both blue- and green-highlighted cells) whose $p<0.1$. The interpretation is that at least one FDS-CrtM interaction term is significant (meaning that its coefficient is statistically different from zero) at $\alpha=0.1$ for each model, implying that the matching of FDS and CrtM variants is important for determining the resulting distribution of carotenoid backbone titers.

Notes for **Supplementary Table 4**:

1. *p*-values below 0.1 are highlighted in gray.
2. Regressions took the matrix algebraic form $T = X\beta + W\gamma + \varepsilon$, where:
   - T is the 64×1 matrix of titers of a carotenoid backbone for each FDS-CrtM combination in the experiment.
   - X constitutes the 64×7 design matrix for the experiment, containing a first column of ones for the constant and 6 columns of predictor variables whose values are either 0 or 1 depending on whether that amino acid substitution (in the FDS or the CrtM) is absent or present in the combination.
   - β is the 7×1 matrix of first-order term coefficients (plus the constant term) to be solved for by the regression.
   - W is the 64×15 design matrix of 2-body (epistatic) interactions whose values are either 0, if both substitutions specified by the entry are not present in the FDS-CrtM combination, or 1 if both are present in the combination.
   - γ is the 15×1 matrix of 2-body interaction term coefficients to be solved for by the regression.
   - ε is the 64×1 matrix of error terms.

Supplementary Note 6.
How *metabolic filtering* works – An Illustrative Model

To date, we have experimentally biosynthesized eight different carotenoid backbone structures, C_{30} ($C_{15}PP$+$C_{15}PP$), C_{35} ($C_{20}PP$+$C_{15}PP$), C_{40} ($C_{20}PP$+$C_{20}PP$), asymmetric C_{40} ($C_{25}PP$+$C_{15}PP$), C_{45} ($C_{25}PP$+$C_{20}PP$), C_{50} ($C_{25}PP$+$C_{25}PP$), C_{55} ($C_{30}PP$+$C_{25}PP$), and C_{60} ($C_{30}PP$+$C_{30}PP$). Co-expression of an FDS variant and a CrtM variant yields some distribution of these eight compounds. The specificity of this two-member pathway (i.e., its product distribution) is, at first approximation, determined by the relative production rate of each of the eight carotenoids C_j. These rates are proportional both to the concentrations of isoprenyl diphosphates generated by the FDS (its product specificity) and the kinetic preference (substrate specificity) of the carotenoid synthase. Because of this relationship, improvements to both factors would multiplicatively alter the distribution of carotenoid backbones.

The following model, intended for illustrative and explanatory purposes (not for fitting to experimental measurements), shows quantitatively how modest improvements in the specificities of successive pathway enzymes can be combined to give substantial focusing of pathway flux to a desired product by "filtering out" undesired precursors from being incorporated into carotenoids.

Assumptions:

1. Each carotenoid C_j is the condensation product of two isoprenyl diphosphates $C_{i1}PP$ and $C_{i2}PP$, which may be identical or different from each other.
2. The product distribution of carotenoid backbones (for a given pairing of FDS- and CrtM- variants) is determined by the production rates (p_j) of each possible backbone.
3. The production rate (p_j) of each carotenoid C_j is determined by the concentrations of the two prenyl diphosphate substrates ($C_{i1}PP$ and $C_{i2}PP$) multiplied by the rate constant for their condensation by a carotenoid synthase variant (k_j). The concentration of synthase enzyme is assumed to be constant across all cases and "included" in the values of k_j. Thus, differences in k_j only reflect differences in the specificity for the various substrates.

Therefore,

$$p_j = k_j \times y_{i1} \times y_{i2} \times I_{Tot}^2$$

Where: y_{i1} and y_{i2} are the molar fractions of isoprenyl diphosphates used to make carotenoid C_j and I_{Tot} is the total concentration of isoprenyl diphosphates. I_{Tot} is assumed to be constant (and equal to 1) for the purposes of this exercise.

We illustrate metabolic filtering in **Supplementary Fig. 7** by considering three cases:

1. *Base case:* This is the simple case where the FDS variant produces an equal-proportion mixture of $C_{15}PP$, $C_{20}PP$, $C_{25}PP$, and $C_{30}PP$ and the CrtM variant condenses all of the two-substrate combinations with the same rate constant. In this case, the molar fraction of C_{50} carotenoid is 12.5%.

2.1. *Precursor enrichment:* In this case, the production of $C_{25}PP$ is enriched due to a change in the step-number specificity of the FDS variant. The CrtM variant still has no preference for particular substrates and condenses all pairs of C_iPP with the same rate constant. Due to the increased fraction of $C_{25}PP$ (in this case, 60%), the fraction of C_{50} carotenoids is increased to 50%. However, enrichment of $C_{25}PP$ also increases the proportion of C_{55} carotenoids to 25%.

2.2. *Condensation specificity:* In this case, as in the base case, the FDS variant once again generates an equal mixture of $C_{15}PP$, $C_{20}PP$, $C_{25}PP$, and $C_{30}PP$, but the CrtM variant exhibits enhanced preference for C_{40}-C_{50} synthesis. The fraction of C_{50} carotenoids is moderately increased to ~32%.

3. *Metabolic filtering:* This case combines the precursor enrichment of **Case 2.1** with the condensation specificity increase of **Case 2.2**. Here, the FDS variant generates an increased proportion of $C_{25}PP$, and the CrtM variant exhibits the same increased preference for that precursor as assumed in **Case 2.2**. The predominant unwanted FDS product, $C_{30}PP$ (20%), is a poor substrate for this CrtM variant, which is reflected by the small amounts of its condensation products (0.5% and 0.2% for C_{55} and C_{60} carotenoids, respectively). Thus, $C_{30}PP$ is largely “filtered out” of being incorporated into carotenoids. The rate constants for C_{45}, and C_{50} synthesis are identical for this CrtM variant, but production of the former backbone is limited by the reduced supply of $C_{20}PP$. Thus, as a result of combining precursor enrichment with condensation specificity, the molar fraction of the target C_{50} carotenoid becomes quite high (~90%) because other precursors are “filtered out” at the FDS or CrtM stage.

Supplementary Note 7.
Directed evolution of phytoene desaturase for C_{50} desaturase activity

Directed evolution of CrtI. Prior establishment of a specific C_{50} backbone pathway (**Fig. 4**) was necessary to enable simple visual screening for C_{50} desaturase activity without erroneously evolving the desaturase for improved or altered activity on C_{40} backbone (phytoene, **1**), the native substrate of phytoene desaturase, CrtI (**Supplementary Fig. 8a**). Using error-prone PCR, we created a library of genes encoding point-mutants of CrtI. These variants were cloned into a pUC-based plasmid with an arabinose promoter. The resultant CrtI plasmid library (size ~10^5) was transformed into *E. coli* cells harboring one of the FDS-CrtM variant pairs that selectively produces C_{50} backbone (pAC-$fds_{Y81A,V157A}$-$crtM_{F26A,W38A,F233S}$). After plating the library onto LB-agar and subsequent colony formation (**Fig. 6a**), nitrocellulose membranes were used to transfer the colonies onto fresh LB-agar plates containing 0.2% (w/v) arabinose. Out of the 2000 colonies surveyed, we isolated 8 colonies with an intense red hue. After a second screening of the 6 hits, we chose six CrtI variants for detailed analysis.

Sequence/product analysis of CrtI variants. Sequence analysis (**Supplementary Table 5**) revealed three mutations N304S, I338V, and F339S/L that significantly increase C_{50} desaturase activity. The CrtI variants were co-transformed with pAC-$fds_{Y81A,V157A}$-$crtM_{F26A,W38A,F233S}$ in *E. coli* and the distribution of C_{50} desaturation products was analyzed. $CrtI_{mut2}$ and $CrtI_{mut8}$, which both possess the N304S mutation, exhibited the highest *in vivo* desaturation levels of all the variants (**Supplementary Fig. 8b**).

Site-saturation mutagenesis of N304 residue and analysis. We then performed a site-saturation mutagenesis experiment on position 304 of CrtI using NNK degenerate oligonucleotides (N: equimolar mixture of dA, dG, dC, dT; K: equimolar mixture of dG and dT), and screened for mutants with elevated C_{50} desaturation activity (pink colonies). The N304P mutant appeared to have the highest *in vivo* desaturation activity (**Supplementary Fig. 8d**). Although the accumulation of C_{50}-lycopene (**8**) is not significantly different between $CrtI_{N304S}$ and $CrtI_{N304P}$, the amount of undesaturated C_{50}-phytoene (**7**) was lower in the cells expressing $CrtI_{N304P}$**.** All of the CrtI variants showed wild-type activity in C_{40} pathway (**Supplementary Fig. 8c**). Thus, they acquired C_{50} desaturase activity without compromising their original C_{40} activity.

Supplementary Note 8.
Plasmid construction

The genes and plasmids used in this study are listed in **Supplementary Tables 7 and 8**, respectively. Plasmid maps are shown in **Supplementary Fig. 11**. The plasmids were constructed using standard manipulation techniques, type IIS restriction enzyme assembly (FASTR[22] and GoldenGate[23]), or homology-based assembly (SLIC[24] and one-step SLIC[25]). Primers used for plasmid construction are listed in **Supplementary Table 9**.

Plasmids with the prefix "pUC" are based on the pUC18m vector[21], which has an EcoRI-XbaI-XhoI-ApaI multi-cloning site under a *lac* promoter.

- **pUC-*crtM*** is identical to pUC-*fl-crtM* in ref. 26. Here, the *crtM* gene were inserted into the XbaI/XhoI site of pUC18m. The sequence upstream *crtM* gene is 5'-**TCTAGA**<u>AGGAGG</u>ATTACAAA-3' (XbaI in boldface and ribosome binding site underlined). XhoI is placed immediately downstream the stop codon of *crtM*.
- **pUC-*fds*** is derived from our previous study[27]. Here, the *fds* gene were inserted into the XbaI/XhoI site of pUC18m. The sequence upstream *fds* gene is 5'-**TCTAGA**AGGAGGAGTAAGCG-3'.
- For the construction of **pUC-*crtM*$_{variants}$** ("*crtM*$_{variants}$" refers to wild-type *crtM* and its variants) and **pUC-*fds*$_{variants}$** ("*fds*$_{variants}$" refers to wild-type *fds* and its variants), site-directed mutations were inserted using FASTR method using primers listed in **Supplementary Table 9**.

Plasmids with the prefix "pAC" are based on the pACmod vector[28].

- For the construction of **pAC-*crtM*$_{variants}$** and **pAC-*fds*$_{variants}$**, the genes and promoters (*Plac-crtM*$_{variants}$ or *Plac-fds*$_{variants}$) were PCR-amplified from pUC-*crtM*$_{variants}$ or pUC-*fds*$_{variants}$, respectively, by using the primers "pUCtopAC-SLIC-BamHI-F/R", and cloned into the BamHI site of pACmod by using SLIC method.
- For the construction of **pAC-*fds*$_{variants}$-*idi*,** *Plac-idi* was PCR-amplified from pUC-*idi* (see below) using the primers "pUCtopAC-SLIC-HindIII-F/R", and ligated into the HindIII site of pAC-*fds*$_{variants}$ (SLIC). pUC-*idi* was made by inserting *idi* gene (amplified from *E. coli* DH10B genome) into XbaI/XhoI site of pUC18m using the same RBS sequence with pUC-crtM.
- For the construction of **pAC-*fds*$_{variants}$-*crtM*$_{variants}$**, *Plac-fds*$_{variants}$ was PCR-amplified from pUC-*fds*$_{variants}$ using "pUCtopAC-SLIC-HindIII-F/R" and ligated into the HindIII site of pAC-*crtM*$_{variants}$ (SLIC).
- For the constructions of **pAC-*fds*$_{Y81A,V157A}$-*crtM*$_{F26A,W38A,F233S}$-*idi***, *Plac-idi* was PCR-amplified from pUC-*idi* and ligated into the *Sal*I site of pAC-*fds*$_{Y81A,V157A}$-*crtM*$_{F26A,W38A,F233S}$, using "pUCtopAC-SLIC-SalI-F/R" primers for PCR and assembled by SLIC.
- **pAC-*crtE-crtB-crtI-idi*** was constructed by inserting *Plac-idi* (PCR-amplified from pUC-*idi*) into the *Cla*I site of pAC-*crtE-crtB-crtI* (this plasmid was made by inserting a *Plac-crtE-crtB-crtI* fragment, amplified from pUC-*crtE-crtB-crtI*[21], into the SalI site of pACmod).
- **pAC-*crtE-crtI-idi*** was constructed by inserting a *Plac-idi* PCR fragment into the SalI site of pAC-*crtE-crtI*[6], using "pUCtopAC-SLIC-HindIII-F/R" primers for PCR and assembled by SLIC.
- **pAC-*crtN-idi*** was constructed by inserting a *Plac-idi* PCR fragment (PCR-amplified from pUC-*idi*)

into SalI, and *Plac-crtN* (PCR-amplified from pUC-*crtN*[6]) into BamHI of pACmod by using "pUCtopAC-SLIC-SalI-F/R" and "pUCtopAC-SLIC-BamHI-F/R" primers, respectively, and cloned into pACmod using SLIC.

The plasmids for the downstream enzymes (*crtI, crtY, crtW,* and *crtZ*) and their derivatives are based on pUCara vectors; these vectors were made by replacing the *lac* promoter of pUC18m (from the O_3 operator site to the O_1 operator site) with the *araC* to *araBAD* promoter region from the pBADHisA vector (Invitrogen).

- **pUCara-*crtI*** was constructed by removing the *lac* promoter region from pUC18m-*crtI* (see below) by amplifying this plasmid using primers "pBAD-vecF/R", where the *araBAD* promoter region amplified from pBADHisA using "pBAD-insF/R" primers was ligated into. pUC18m-*crtI* was originally constructed by amplifying *crtI* gene using the primers "crtI-F/R" and cloned into XhoI/ApaI site of pUC18m. If necessary, the other *crtI* variants were cloned into the XhoI/ApaI site of pUCara-*crtI*.
- **pUCara-*crtI-crtY*** was constructed by the FASTR assembly of the following two PCR-amplified fragments: (1) the entire pUCara-*crtI* sequence using the primers "crtIdownstream-F/R" (inverse PCR) and (2) amplification of the *crtY* gene using the primers "crtY-F/R". The resultant construct has SpeI site downstream *crtY*.
- **pUCara-*crtI-crtY-crtW-crtZ*** was constructed by digesting the codon-optimized *crtW-crtZ* operon (purchased from DNA2.0, Menlo Park, Calif., see below for the sequence) using XbaI (which flanks the whole sequence), and ligating into the *Spe*I site of pUCara-$crtI_{variants}$-*crtY*. The final construct is: XhoI-*crtI*-ApaI-*crtY*-SpeI-*crtW*-ClaI-*crtZ*-HindIII.
- **pUCara-*crtI-crtY-crtW*** was constructed by digesting pUCara-*crtI-crtY-crtW-crtZ* with ClaI and HindIII, filling the resultant overhang by T4 DNA polymerase, followed by intra-molecular blunt-end ligation.
- **pUCara-*crtI-crtY-crtZ*** was constructed by digesting pUCara-*crtI-crtY-crtW-crtZ* with SpeI and ClaI, filling the overhang using T4 DNA polymerase, and ligating the resultant blunt ends.

pET-$fds_{variants}$ were constructed by amplifying the *fds* gene or its variants by adding the sequence 5'-CCATGGgcagcagccatcatcatcatcatcacagcGGATCC-3' upstream of the second codon of *fds*, and then inserting it into the NcoI/XhoI site of pET15b.

Codon-optimized crtW-crtZ operon sequence (ORF in lowercase, restriction enzyme sequence in bold):

T**TCTAGA**TA**ACTAGT**AGGAGGATTACAAAatgaccgcagctgtcgcagaacctcgcattgtaccgcg
ccaaacctggatcggcctgaccctggcgggtatgattgtggcgggctggggttctctgcacgtgtac
ggtgtgtacttccaccgttggggcaccagcagcctggttatcgtcccggctatcgtggccgttcaga
cgtggttgtcggttggcctgtttattgtcgcacatgacgccatgcacggttccctggccccaggccg
tccgcgcctgaacgcagcggtgggtcgtctgacgctgggtctgtatgcaggcttccgtttcgatcgc
ttgaaaacggcgcaccacgcgcatcatgcggctccgggtaccgcagatgacccggacttttacgcgc
ctgcgccacgcgccttcctgccgtggtttttgaactttttccgtacctatttcggttggcgcgagat
ggcggttctgaccgcgctggtcctgatcgcgctgtttggcttgggtgcccgtccggcgaatctgttg

acttttgggccgcaccggcgctgctgagcgcgctgcaactgttcacgtttggcacctggctgccgc
accgtcacacggaccagccgttcgcggatgctcatcatgcacgcagcagcggttatggtccggttct
gagcctgctgacctgctttcatttcggtcgtcatcacgagcaccacctgacgccgtggcgtccgtgg
tggcgtttgtggcgtggtgaaagctaa**ATCGAT**TCACTGTATAACATTAAGAAGGAGGATTACAAAa
tggcatggctgacctggatcgcactgttcctgaccgcattcctgggtatggaggctttcgcgtggat
catgcaccgttatgtcatgcacggtttcttgtggtcgtggcatcgtagccatcacgagccgcacgac
cacccgctggaaaagaacgacctgtttgccgttgtctttgccgctccggcgattgttatggtggcgg
tgggtctgcacctgtggccttgggccttgccggtcggtctgggtattactgcgtacggcatggttta
cttcttctttcatgatggcctggtgcatcgtcgtttcccgacgggctttagcggtcgcagcggcttt
tggacccgtcgcatccaggcgcaccgtctgcaccatgcagtccgcacgcgtgagggctgcgtgtcct
ttggcttcttgtgggttcgcagcgcgcgtgccctgaaagcggaactggcgcaaaaacgcggtagcag
cagctctggtgcataa**AAGCTT**TA**TCTAGA**A

Supplementary References

1. Tachibana, A. A novel prenyltransferase, farnesylgeranyl diphosphate synthase, from the haloalkaliphilic archaeon, *Natronobacterium pharaonis*. *FEBS Lett.* **341**, 291-294 (1994).
2. Tachibana, A. et al. Novel prenyltransferase gene encoding farnesylgeranyl diphosphate synthase from a hyperthermophilic archaeon, *Aeropyrum pernix*. Molecularevolution with alteration in product specificity. *Eur. J. Biochem.* **267**, 321-328 (2000).
3. Ogawa, T., Yoshimura, T. & Hemmi, H. Geranylfarnesyl diphosphate synthase from *Methanosarcina mazei*: Different role, different evolution. *Biochem. Biophys. Res. Commun.* **393**, 16-20 (2010).
4. Tobias, A.V. Directed evolution of biosynthetic pathways to carotenoids with unnatural carbon backbones. *Doctoral Dissertation, California Institute of Technology,* http://resolver.caltech.edu/CaltechETD:etd-08232005-174620 (2005).
5. Ohnuma, S. et al. A role of the amino acid residue located on the fifth position before the first aspartate-rich motif of farnesyl diphosphate synthase on determination of the final product. *J. Biol. Chem.* **271**, 30748-30754 (1996).
6. Umeno, D. & Arnold, F.H. Evolution of a pathway to novel long-chain carotenoids. *J. Bacteriol.* **186**, 1531-6 (2004).
7. Tobias, A.V. & Arnold, F.H. Biosynthesis of novel carotenoid families based on unnatural carbon backbones: a model for diversification of natural product pathways. *Biochim. Biophys. Acta Mol. Cell Biol. Lipids* **1761**, 235-246 (2006).
8. Britton, G. Overview of carotenoid biosynthesis. in *Carotenoids, vol. 3: Biosynthesis and Metabolism* (eds. Britton, G., Liaaen-Jensen, S. & Pfander, H.) 13-147 (Birkhauser Verlag, Basel, Switzerland, 1998).
9. Takaichi, S. et al. The carotenoid 7,8-dihydro-ψ end group can be cyclized by the lycopene cyclases from the bacterium *Erwinia uredovora* and the higher plant *Capsicum annuum*. *Eur. J. Biochem.* **241**, 291-296 (1996).
10. Choi, S.K., Matsuda, S., Hoshino, T., Peng, X. & Misawa, N. Characterization of bacterial β-carotene 3,3'-hydroxylases, CrtZ, and P450 in astaxanthin biosynthetic pathway and adonirubin production by gene combination in *Escherichia coli*. *Appl. Microbiol. Biotechnol.* **72**, 1238-1246 (2006).
11. Choi, S.K. et al. Characterization of β-carotene ketolases, CrtW, from marine bacteria by complementation analysis in *Escherichia coli*. *Mar. Biotechnol.* **7**, 515-522 (2005).
12. Karrer, P. & Eugster, C.H. Synthesen von Carotinoiden VI. Synthese eines Homologen des β-Carotins mit 15 konjugierten Doppelbindungen: Decapreno-β-carotin. *Helv. Chim. Acta* **34**, 28-33 (1951).
13. Milon, A., Wolff, G., Ourisson, G. & Nakatani, Y. Organization of carotenoid-phospholipid bilayer systems. Incorporation of zeaxanthin, astaxanthin, and their C_{50} homologues into dimyristoylphosphatidylcholine vesicles. *Helv. Chim. Acta* **69**, 12-24 (1986).
14. Krubasik, P., Kobayashi, M. & Sandmann, G. Expression and functional analysis of a gene cluster involved in the synthesis of decaprenoxanthin reveals the mechanisms for C_{50} carotenoid formation. *Eur. J. Biochem.* **268**, 3702-3708 (2001).
15. Ohnuma, S. et al. A pathway where polyprenyl diphosphate elongates in prenyltransferase. Insight into a common mechanism of chain length determination of prenyltransferases. *J. Biol. Chem.* **273**, 26705-26713 (1998).

16. Sandmann, G. Evolution of carotene desaturation: the complication of a simple pathway. *Arch. Biochem. Biophys.* **483**, 169-174 (2009).
17. Umeno, D. & Arnold, F.H. A C_{35} carotenoid biosynthetic pathway. *Appl. Environ. Microbiol.* **69**, 3573-3579 (2003).
18. Umeno, D., Tobias, A.V. & Arnold, F.H. Diversifying carotenoid biosynthetic pathways by directed evolution. *Microbiol. Mol. Biol. Rev.* **69**, 51-78 (2005).
19. Fraser, P.D., Shimada, H. & Misawa, N. Enzymic confirmation of reactions involved in routes to astaxanthin formation, elucidated using a direct substrate in vitro assay. *Eur. J. Biochem.* **252**, 229-236 (1998).
20. Tao, L., Schenzle, A., Odom, J.M. & Cheng, Q. Novel carotenoid oxidase involved in biosynthesis of 4,4'-diapolycopene dialdehyde. *Appl. Environ. Microbiol.* **71**, 3294-3301 (2005).
21. Umeno, D., Tobias, A.V. & Arnold, F.H. Evolution of the C_{30} carotenoid synthase CrtM for function in a C_{40} pathway. *J. Bacteriol.* **184**, 6690-6699 (2002).
22. Kotera, I. & Nagai, T. A high-throughput and single-tube recombination of crude PCR products using a DNA polymerase inhibitor and type IIS restriction enzyme. *J. Biotechnol.* **137**, 1-7 (2008).
23. Engler, C., Kandzia, R. & Marillonnet, S. A one pot, one step, precision cloning method with high throughput capability. *PLoS One* **3**, e3647 (2008).
24. Li, M.Z. & Elledge, S.J. Harnessing homologous recombination *in vitro* to generate recombinant DNA via SLIC. *Nat. Methods* **4**, 251-256 (2007).
25. Jeong, J.Y. et al. One-step sequence- and ligation-independent cloning as a rapid and versatile cloning method for functional genomics studies. *Appl. Environ. Microbiol.* **78**, 5440-5443 (2012).
26. Furubayashi, M., Saito, K. & Umeno, D. Evolutionary analysis of the functional plasticity of *Staphylococcus aureus* C_{30} carotenoid synthase. *J. Biosci. Bioeng.* **4**, 431-436 (2014).
27. Furubayashi, M. et al. A high-throughput colorimetric screening assay for terpene synthase activity based on substrate consumption. *PLoS One* **9**, e93317 (2014).
28. Schmidt-Dannert, C., Umeno, D. & Arnold, F.H. Molecular breeding of carotenoid biosynthetic pathways. *Nat. Biotechnol.* **18**, 750-753 (2000).
